# A network meta‐analysis of association between cardiometabolic risk factors and COVID‐19 outcome severity

**DOI:** 10.1111/1753-0407.13445

**Published:** 2023-08-30

**Authors:** Alina Binbin Li, Bo Yang, Yufei Li, Rachel Huynh, Samuel Shim, Kenneth Lo, Jie Li, Andrew Zullo, Wen‐Chih Wu, Simin Liu

**Affiliations:** ^1^ Department of Epidemiology, School of Public Health Brown University Providence Rhode Island USA; ^2^ Centre for Global Cardiometabolic Health, Departments of Epidemiology, Medicine, and Surgery Brown University Providence Rhode Island USA; ^3^ Department of Applied Biology and Chemical Technology The Hong Kong Polytechnic University Kowloon Hong Kong China; ^4^ Guangdong Cardiovascular Institute, Guangdong Provincial People's Hospital Guangdong Academy of Medical Sciences Guangzhou China; ^5^ Global Health Research Center, Guangdong Provincial People's Hospital Guangdong Academy of Medical Sciences Guangzhou China; ^6^ Division of Cardiology, Veterans Affairs Medical Center and The Miriam Hospital, Department of Medicine Alpert Medical School Providence Rhode Island USA

**Keywords:** cardiometabolic risk factors, COVID‐19, network meta‐analysis, 心血管代谢危险因素, 2019冠状病毒病, 网络*meta*分析

## Abstract

**Background:**

Cardiometabolic comorbidities have been associated with a higher risk of COVID‐19 severity and mortality, but more investigations are needed to determine which comorbidity is more detrimental.

**Methods:**

Embase, Emcare, and MEDLINE were searched systematically for prospective and retrospective studies assessing the associations of cardiometabolic risk factors and COVID‐19 outcomes of hospitalization, severity, and mortality among COVID‐19‐diagnosed patients. Literature search was performed from first publication to May 19, 2021. Study quality was assessed by the Newcastle‐Ottawa Scale.

**Results:**

From the literature search, 301 studies suggested that all included cardiometabolic risk factors were associated with a higher risk of COVID‐19 hospitalization, severity, and mortality, except that overweight was associated with a decreased risk of mortality (relative risk [RR] 0.88; 95% CI, 0.80–0.98). Patients with diabetes (RR 1.46; 95% CI, 1.45–1.47) were most likely to be hospitalized; patients with heart failure had the highest risk for severe COVID‐19 outcomes (RR 1.89; 95% CI, 1.71–2.09); while patients with stroke were most susceptible to overall mortality (RR 1.99; 95% CI, 1.90–2.08). In the network meta‐analysis, cerebrovascular disease had the highest impact (RR 1.69; 95% CI, 1.65–1.73) on COVID‐19 outcomes compared to other cardiometabolic risk factors. For different combinations of risk factors, cardiovascular disease and diabetes combined (RR 6.98; 95% CI, 5.28–9.22) was more detrimental than others.

**Conclusions:**

Considering the high prevalence of cardiometabolic comorbidities and risk of all severe outcomes, patients with cardiometabolic comorbidities should be prioritized in vaccination and treatment development of COVID‐19.

## Graphical abstract

**Highlights**

- Among 301 studies, diabetes mellitus was the most studied cardiometabolic risk factor.
- In the network meta‐analysis, cerebrovascular disease had the highest impact (relative risk [RR] 1.69; 95% CI, 1.65–1.73) on COVID‐19 outcomes compared to other cardiometabolic risk factors.
- For different combinations of risk factors, cardiovascular disease and diabetes mellitus combined (RR 6.98; 95% CI, 5.28–9.22) was more detrimental than others.

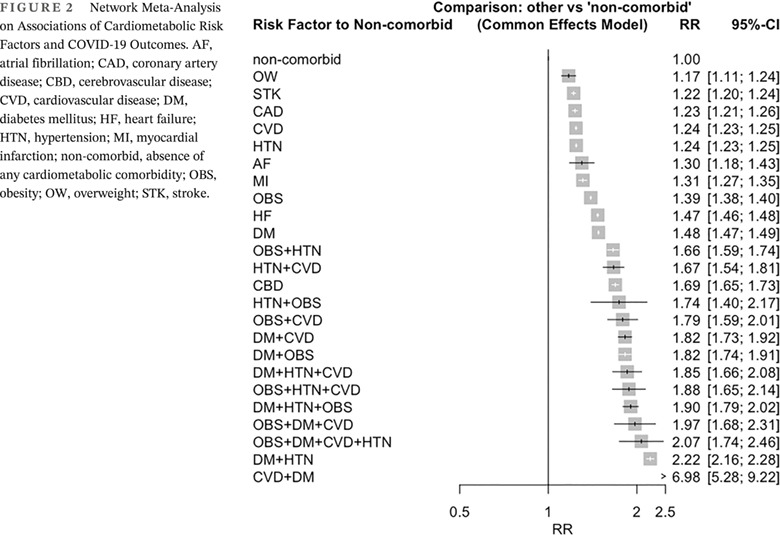

## 摘要

背景:心血管代谢疾病已被证实与COVID‐19的严重程度和死亡率增加有关, 但需要进一步研究来确定哪种疾病更具危害性。

方法:对Embase、Emcare和MEDLINE®进行系统检索, 评估心血管代谢危险因素与COVID‐19患者住院、严重程度和死亡率之间的关联。文献检索从首次发表到2021年5月19日。研究质量由纽卡斯尔‐渥太华量表评估。

结果:从文献检索中, 共有301项研究表明, 除超重与死亡率降低的风险相关(RR 0.88, 95% CI:0.80至0.98)外, 所有包括的心血管代谢危险因素都与COVID‐19住院、严重程度和死亡率增加的风险相关。糖尿病患者最有可能住院(RR 1.46, 95% CI:1.45至1.47), 心力衰竭患者最容易出现严重COVID‐19结果(RR 1.89, 95% CI:1.71至2.09), 而卒中患者最容易发生总体死亡率(RR 1.99, 95% CI:1.90至2.08)。在网络*meta*分析中, 与其他心血管代谢危险因素相比, 脑血管疾病对COVID‐19结果的影响最大(RR 1.69, 95% CI:1.65至1.73)。对于不同的危险因素组合, 心血管疾病和糖尿病的组合(RR 6.98, 95% CI:5.28至9.22)比其他组合更具危害性。

结论:考虑到心血管代谢疾病的高发病率和所有严重结果的风险, 患有心血管代谢疾病的患者应优先接种疫苗并参与COVID‐19的治疗。

## 1 INTRODUCTION

COVID‐19 caused by SARS‐CoV‐2 was first reported in December 2019^1^ and spread rapidly worldwide, leading to an ongoing pandemic. It can infect people of all age groups, although severe symptoms are more common in older adults.^2^ Infected patients present a wide range of clinical manifestations and outcomes, including subclinical infections, hospitalization, organ dysfunction, and death.^3^

Nearly half of the patients with COVID‐19^2^, ^3^, ^4^ have comorbidities, including obesity, hypertension (HTN), diabetes mellitus (DM), and cardiovascular diseases (CVD).^5^, ^6^ Patients with underlying cardiometabolic comorbidities are more likely to have severe symptoms and get admitted to the intensive care unit (ICU).^4^ Moreover, the percentages of patients with cardiometabolic comorbidities are much higher among the fatality cases than survival cases.^7^ Much of the prior research conducted has revealed associations between single underlying comorbidities and COVID‐19 outcomes.^7^, ^8^, ^9^, ^10^, ^11^, ^12^, ^13^, ^14^, ^15^, ^16^ Studies have also been done to analyze the effects of combinations of comorbidities toward the adverse outcomes of COVID‐19.^17^, ^18^, ^19^, ^20^, ^21^ Inconsistencies in the associations were observed among the publications, and definitive conclusions have yet to be made. A rapid increase in systematic reviews and meta‐analyses has provided a great summary of the published papers^22^, ^23^, ^24^, ^25^, ^26^; however, due to the methodological restrictions of meta‐analyses,^27^ different cardiometabolic risk factors of COVID‐19 cannot be compared with one another because of the heterogeneity among the studies.

To compare and systematically evaluate the associations of cardiometabolic risk factors with COVID‐19 incidence and adverse outcomes, we conducted a quantitative network meta‐analysis treating multiple risk factors simultaneously as if they were from a network of studies connected by both direct and indirect evidence.^28^, ^29^ The impact of individual and combined cardiometabolic risk factors toward COVID‐19 outcomes was subsequently assessed to rank the relative importance of these influential risk factors.

## 2 METHODS

### 2.1 Search strategy

This systematic review and meta‐analysis were conducted following the Preferred Reporting Items for Systematic Reviews and Meta‐analyses (PRISMA) Statement and Agency for Healthcare Research and Quality (AHRQ) guidelines. The search terms consisted of subject headings and keywords for cardiometabolic risk factors and study population. Risk factors of interest included atrial fibrillation (AF), coronary artery disease (CAD), cerebrovascular disease (CBD), CVD, DM, heart failure (HF), HTN, myocardial infarction (MI), obesity, overweight, and stroke. Outcomes of interest included hospitalization and length of stay; disease severity as measured by days in emergency room, days in ICU, days on ventilator, and complications—blood clots (e.g., stroke, heart attack, pulmonary embolism, deep vein thrombosis), kidney failure/dialysis, and mortality. The study population was restricted to patients diagnosed with COVID‐19.

The literature search was performed by two researchers (K. Lo and S. Liu) in three databases with a language restriction of English and Chinese: (1) Embase 1910 to May 19, 2021; (2) Ovid Emcare 1995 to 2021 Week 21; and (3) Ovid MEDLINE and Epub Ahead of Print, In‐Process & Other Non‐Indexed Citations 1946 to May 19, 2021. Studies that were (a) literature reviews, letters, and abstracts from conference proceedings; (b) providing insufficient data information; or (c) duplicate publications were excluded. The full search strategy is presented in Table S1.

### 2.2 Study selection

**FIGURE 1:**
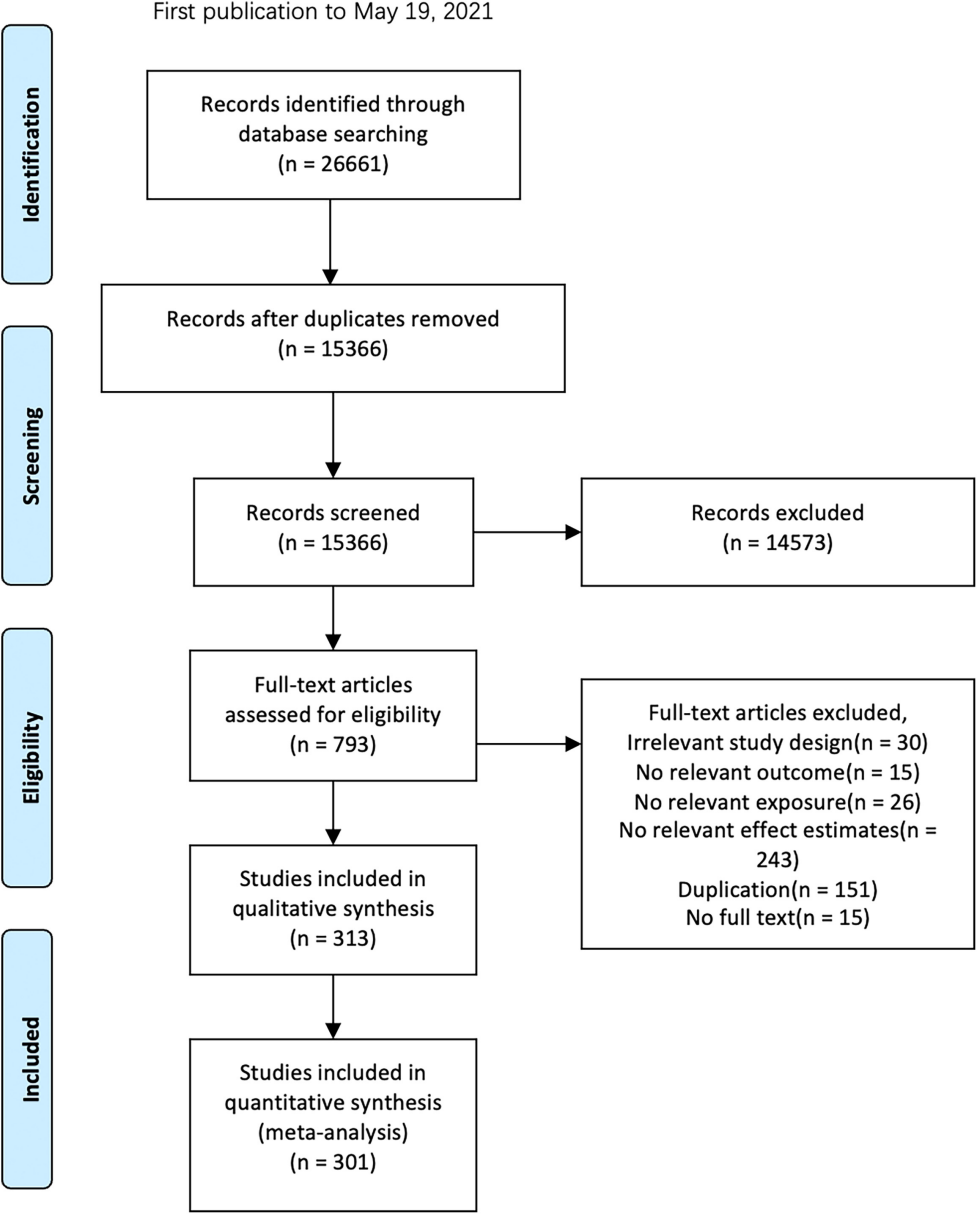
Search and selection process by timeframe according to the Preferred Reporting Items for Systematic Reviews and Meta‐Analyses (PRISMA) checklist.

All study information retrieved from the literature search was downloaded into Microsoft Excel. After duplication removal, three researchers performed abstract screening and full‐text screening (B. Yang, Y. Li, and A. Li). Disagreements were resolved by a third investigator (K. Lo) or by consensus. Only primary studies that provided the required association information between cardiometabolic risk factors and COVID‐19‐related outcomes were included in our study. A total number of 301 studies were included in the meta‐analyses (Figure 1).

### 2.3 Data extraction and quality assessment

The three investigators performing the literature selection extracted the data from the included studies. A data extraction form was developed for this project and extracted the following information: author, date, country, number of males, age, sample size, comorbidities, basis of disease severity, and the effect size estimates. The assessment of literature quality was performed by four investigators (B. Yang, Y. Li, S. Shim, and R. Huynh) following the Newcastle‐Ottawa Scale (NOS). Disagreements were resolved by a third investigator or by consensus. The NOS evaluate the quality of a study according to three aspects: selection, outcome, and comparability. Higher NOS scores represent higher literature quality, where a maximum of nine can be given. NOS scores of all included studies can be found in Table S3.

### 2.4 Data synthesis and analysis

A conventional meta‐analysis and a network meta‐analysis were performed using R version 3.6.2. The relative risk (RR) and relevant 95% CI calculated from the fixed effect models were used to estimate the pooled results from included studies. Statistical heterogeneity across included studies was investigated using the inconsistency index (*I* ^2^), with *I* ^2^ of at least 50% indicating moderate heterogeneity and *I* ^2^ ≥ 75% indicating high heterogeneity. We further conducted a sensitivity analysis by restricting the meta‐analysis to studies with a sample size larger than 1000. This is to reduce the heterogeneity between studies and identify potential exaggerated effects of the small studies on the pooled estimates.

The ranking of combined and independent effects of cardiometabolic risk factors toward COVID‐19 outcomes was assessed through the network meta‐analysis approach. The risk factor ranking was computed by means of the P‐scores, which measure the certainty that one risk factor is more influential than another. The evidence network was illustrated to visualize the network geometry and node connectivity in network plots. The comparative effects of the risk factors were calculated using a multivariate fixed effect meta‐analysis model. Q statistics both within designs and between designs in the model were used to evaluate the heterogeneity across studies.

## 3 RESULTS

A total of 26 661 studies were identified through the literature search, and 301 of them were included in the qualitative synthesis and meta‐analyses after the abstract and full‐text screening (Figure 1). Most studies reported COVID‐19 outcomes of disease severity (*n* = 402) and mortality (*n* = 605), but fewer reported hospitalizations (*n* = 120) (Table S2). DM was the most studied cardiometabolic risk factors for COVID‐19 (*n* = 334), followed by HTN (*n* = 254), while AF was the least studied condition (*n* = 13) (Table S2). The study qualities were rated in the NOS. Of the 301 studies, 241 studies were of high, 59 of moderate, and 1 of low quality (Table S3).

### 3.1 Conventional meta‐analysis on cardiometabolic risk factors

As shown in Table 1, all cardiometabolic risk factors were associated with an increased risk of hospitalization. These associations were all statistically significant except for CAD (RR 1.07; 95% CI, 0.99–1.15). Patients with DM had the highest risk of hospitalization due to COVID‐19 compared to all other risk factors (RR 1.46; 95% CI, 1.45–1.47). Patients with HF or AF also had more than 40% higher risk of hospitalization compared to those without (1.43 [95% CI, 1.41–1.44] and 1.40 [95% CI, 1.22–1.61], respectively). MI and overweight were not synthesized since there were not enough studies.

**TABLE 1:** Effect estimates of associations between cardiometabolic risk factors and hospitalization, disease severity, and overall mortality in COVID‐19 patients.

	Hospitalization RR (95% CI)	Disease severity RR (95% CI)	Overall mortality RR (95% CI)
Atrial fibrillation	1.40 (1.22–1.61)	NA	1.22 (1.07–1.40)
Coronary artery disease	1.07 (0.99–1.15)	1.50 (1.38–1.64)	1.23 (1.20–1.25)
Cerebrovascular disease	1.17 (1.03–1.34)	1.24 (1.07–1.43)	1.72 (1.67–1.77)
Cardiovascular disease	1.23 (1.22–1.25)	1.33 (1.31–1.36)	1.20 (1.17–1.23)
Diabetes mellitus	1.46 (1.45–1.47)	1.42 (1.39–1.45)	1.50 (1.49–1.51)
Heart failure	1.43 (1.41–1.44)	1.89 (1.71–2.09)	1.65 (1.62–1.69)
Hypertension	1.25 (1.23–1.26)	1.26 (1.22–1.31)	1.25 (1.23–1.26)
Myocardial infarction	NA	NA	1.21 (1.16–1.26)
Obesity	1.35 (1.34–1.37)	1.48 (1.45–1.52)	1.41 (1.39–1.42)
Overweight	NA	1.33 (1.22–1.44)	0.88 (0.80–0.98)
Stroke	1.13 (1.11–1.15)	1.61 (1.27–2.05)	1.99 (1.90–2.08)

*Note*: Estimates based on fewer than three studies were not included. Estimates were computed based on pooled analysis of fixed effects model. Disease severity was measured by days in emergency room, days in intensive care unit, days on ventilator, and complications—blood clots (e.g., stroke, heart attacks, pulmonary embolism, deep vein thrombosis), and kidney failure/dialysis.

Abbreviations: NA, not applicable; RR, relative risk.

As for COVID‐19 disease severity, patients with HF had the highest risk (RR 1.89; 95% CI, 1.71–2.09), followed by stroke (RR 1.61; 95% CI, 1.27–2.05) and CAD (RR 1.50; 95% CI, 1.38–1.64). Compared to hospitalization, the pooled estimates of the associations between each cardiometabolic risk factor and disease severity were all increased, except for DM which had a 4% lower risk and a wider CI. AF and MI were not analyzed due to the limited number of studies.

Over half of the publications were on overall mortality, and we were able to identify enough studies of each cardiometabolic risk factor for meta‐analysis. Among all the included cardiometabolic conditions, stroke and CBD were the most significant risk factors for COVID‐19‐related mortality. Patients with stroke had almost two‐fold the risk of mortality compared to those without stroke (RR 1.99; 95% CI, 1.90–2.08). CBD was found to increase the risk of mortality by over 70% (RR 1.72; 95% CI, 1.67–1.77). All other cardiometabolic risk factors were associated with an increased risk of mortality except overweight. It was shown that overweight was associated with a 12% decrease of mortality risk (RR 0.88; 95% CI, 0.80–0.98).

Comparing the associations between each cardiometabolic risk factor and the three outcomes, it was found that DM (RR 1.46, 1.42, and 1.50), HTN (RR 1.25, 1.26, and 1.25), and obesity (RR 1.35, 1.48, and 1.41) had consistent effects on hospitalization, disease severity, and mortality (Table 1). It was surprising that the effects of obesity and DM on the outcomes were almost comparable. Other cardiometabolic risk factors had more varied effects on the outcomes, with stroke showing the most drastic differences in its effects on outcomes (RR: hospitalization 1.13, disease severity 1.61, and mortality 1.99).

### 3.2 Network analyses

**FIGURE 2:**
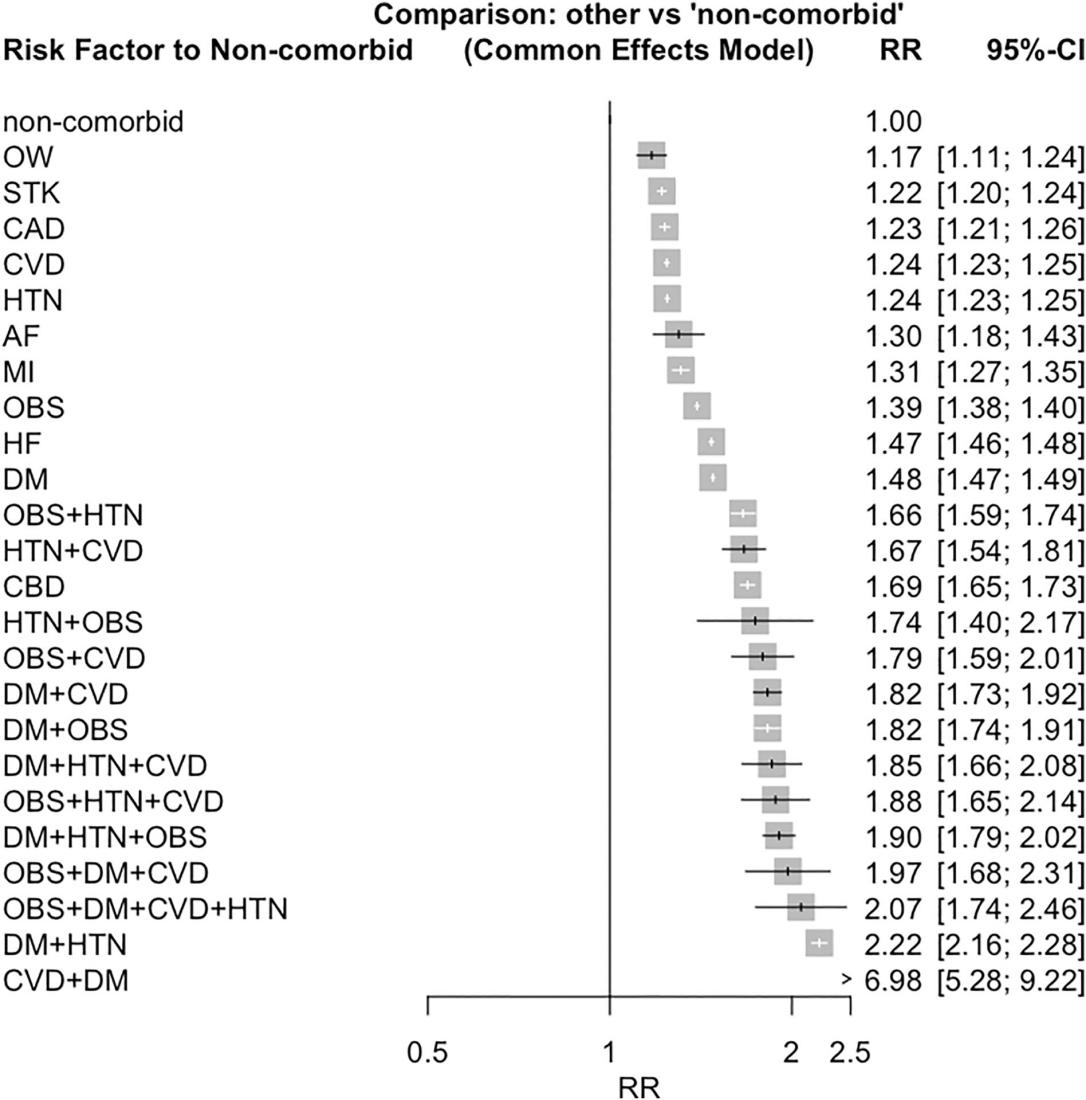
Network Meta‐Analysis on Associations of Cardiometabolic Risk Factors and COVID‐19 Outcomes. AF, atrial fibrillation; CAD, coronary artery disease; CBD, cerebrovascular disease; CVD, cardiovascular disease; DM, diabetes mellitus; HF, heart failure; HTN, hypertension; MI, myocardial infarction; non‐comorbid, absence of any cardiometabolic comorbidity; OBS, obesity; OW, overweight; STK, stroke.

Hospitalizations, severe outcomes, or mortality due to COVID‐19 were combined as one composite outcome and studied in a network analysis of all data accrued (*n* = 301). Diabetes, HTN, and obesity had the greatest number of studies on risk of COVID‐19 outcomes (Figure S1). The ranking of any cardiometabolic risk factor affecting hospitalization, severe outcomes, or mortality was led by CBD (RR 1.69; 95% CI, 1.65–1.73). DM, HF, and obesity each had at least a 35% greater risk of the composite COVID‐19 outcome (Figure 2). Combined risk factors, such as DM + HTN, all had higher impacts toward the outcome than individual risk factors with a minimum of 1.66 times the risk. CVD together with DM had the greatest RR estimates of 6.98 (95% CI, 5.28–9.22). Possible publication bias was indicated by the funnel plot (Figure S2; *p* = 0.0113), and a moderate amount of heterogeneity was suggested across studies likely due to the varied methodology and exposures studied (*I* ^2^ ≥ 50%).

When analyzing the outcomes one at a time, the rankings of cardiometabolic factors were different from each other. For hospitalization (Figure S3), combined risk factors were the most influential ones, with DM + HTN having the highest risk (RR 2.60; 95% CI, 2.04–3.31). When comparing single risk factors, DM had the highest ranking in hospitalization risk, while CAD had the lowest. The most detrimental combination of cardiometabolic risk factors for COVID‐19 severity was HTN + CVD (RR 4.52; 95% CI, 2.59–7.89; Figure S4). The ranking of single cardiometabolic risk factors was led by HF (RR 1.89; 95% CI, 1.71–2.08), stroke, and CAD. More reports were on mortality, where CVD + DM was the most significant risk factor (RR 6.89; 95% CI, 5.28–9.22; Figure S5). Stroke had the highest ranking among all cardiometabolic risk factors and was more detrimental than a large proportion of the cardiometabolic risk factor combinations (RR 1.99; 95% CI, 1.90–2.08). For all three outcomes, the heterogeneity among studies in the network meta‐analysis was substantial (*I* ^2^ ≥ 75%).

### 3.3 Sensitivity analyses

The included studies were restricted to those with a sample size larger than 1000 in the sensitivity analyses. Although some of the pooled estimates changed, the differences were very small (Table 2). The effect estimates in the sensitivity and main analysis were comparable. Studies with small sample size did not significantly affect the pooled estimates in the meta‐analyses.

**TABLE 2:** Effect estimates of associations between cardiometabolic risk factors and hospitalization, disease severity, and overall mortality in COVID‐19 patients in studies with a sample size larger than 1000.

	Hospitalization RR (95% CI)	Disease severity RR (95% CI)	Overall mortality RR (95% CI)
Atrial fibrillation	1.40 (1.22–1.61)	NA	1.24 (1.08–1.43)^a^
Coronary artery disease	1.07 (0.99–1.15)	1.50 (1.37–1.64)^a^	1.24 (1.22–1.27)^a^
Cerebrovascular disease	1.17 (1.03–1.34)	1.24 (1.07–1.43)	1.72 (1.67–1.77)
Cardiovascular disease	1.23 (1.22–1.25)	1.33 (1.31–1.36)	1.19 (1.16–1.22)^a^
Diabetes mellitus	1.46 (1.45–1.47)	1.41 (1.37–1.44)^a^	1.50 (1.49–1.51)
Heart failure	1.43 (1.41–1.44)	1.91 (1.73–2.11)^a^	1.65 (1.62–1.69)
Hypertension	1.25 (1.23–1.26)	1.25 (1.21–1.30)^a^	1.27 (1.25–1.28)^a^
Myocardial infarction	NA	NA	1.21 (1.16–1.26)
Obesity	1.35 (1.34–1.37)	1.48 (1.45–1.51)^a^	1.41 (1.39–1.42)
Overweight	NA	1.33 (1.22–1.45)^a^	0.87 (0.79–0.97)^a^
Stroke	1.13 (1.11–1.15)	1.64 (1.25–2.15)^a^	2.11 (2.01–2.21)^a^

*Note*: Estimates based on fewer than three studies were not included. Estimates were computed based on pooled analysis of fixed effects model. Disease severity was measured by days in emergency room, days in intensive care unit, days on ventilator, complications—blood clots (e.g., stroke, heart attacks, pulmonary embolism, deep vein thrombosis), and kidney failure/dialysis.

Abbreviations: NA, not applicable; RR, relative risk.

^a^ Effect estimates in sensitivity analysis are different from those in main analysis.

## 4 DISCUSSION

In this systematic review and quantitative assessment of all published literature concerning cardiometabolic risk factors and COVID‐19 outcomes, we observed strong positive associations between them, except for the combination of obesity and mortality. The significant associations of CBD, DM, obesity, HTN, stroke, and HF with hospitalization, severity, and mortality were consistent with prior reports.

This network meta‐analysis is a comprehensive evaluation of 747 prospective and retrospective studies, 73 928 213 COVID‐19 patients, and multiple cardiometabolic comorbidities. This analysis uniquely compares combinations of 12 cardiometabolic risk factors individually or in combination through network meta‐analysis. Since cardiometabolic diseases are frequently clustered, the network meta‐analysis accounts for comorbidities, study heterogeneity, and indirect associations. Previously, a meta‐analysis including 24 studies on DM, HTN, chronic pulmonary diseases, and CVD found similar association patterns with COVID‐19 adverse outcomes.^30^ There were also two studies that attempted to rank cardiometabolic risk factors with models and reported a different relative ranking of cardiometabolic risk factors than our network analysis.^31^, ^32^ Although heterogeneities existed across studies, CHD, CAD, and CVD appeared to have the largest impact on COVID‐19 mortality and severity, followed by HTN and DM.^30^, ^33^, ^34^ Cardiac injury, stress, and other complications indicated by biomarkers are particularly prevalent among individuals with cardiometabolic disorders, possibly leading to an increased risk of mortality.^35^ Like the strong associations for obesity found in our network analysis, one genome‐wide association study (GWAS) reported body mass index to be strongly associated with COVID‐19 hospitalization.^34^ Underlying genotypic and phenotypic factors related to angiotensin I‐converting enzyme 2 (ACE2) are associated with obesity and DM and may be involved in the mechanisms of COVID‐19 pathogenesis.^36^ Obesity and DM were found to be consistently associated with COVID‐19‐related hospitalization, severity, and mortality in our study. This aligns with prior reports that worse COVID‐19 outcomes were associated with the presence of impaired glucose metabolism.^37^

As the COVID‐19 pandemic continues to rage on, additional or unanticipated factors may affect the associations between cardiometabolic comorbidities and COVID‐19 outcomes, including the recognition of the Omicron and Delta SARS‐CoV‐2 variants and vaccinations that affect COVID‐19 severity.^38^, ^39^, ^40^ It should also be noted that the risk factors for the included cardiometabolic comorbidities can also attribute to the risk of COVID‐19 outcomes. For instance, visceral obesity and characteristics of impaired metabolic health such as hyperglycemia, HTN, and subclinical inflammation are associated with a high risk of severe COVID‐19.^41^ Moreover, recent data further indicate that these factors might also promote vaccine breakthrough COVID‐19 infections in fully vaccinated individuals.^42^ This can be due to the downregulating of the immune responses in patients with cardiometabolic comorbidities. For instance, hyperglycemia and insulin resistance have been found to modulate the body's immune response, potentially driving immunosenescence and contributing to an increased risk of severe COVID‐19.^42^ Future research should explore the differences of risk among populations with different cardiometabolic comorbidities and variants of COVID‐19.

Although we tried to conduct a comprehensive meta‐analysis and included 11 different comorbidities, certain cardiometabolic conditions have still been overlooked, for example, chronic kidney disease (CKD) and liver disease. Emerging evidence suggests that individuals with CKD and liver disease are at a higher risk of severe outcomes when infected with COVID‐19. Several studies have reported a significant association between CKD and adverse clinical outcomes, including higher rates of hospitalization, ICU admission, and mortality in COVID‐19 patients with preexisting kidney disease.^43^, ^44^ CKD has been identified as an independent risk factor for COVID‐19 severity, with decreased kidney function correlating with worse outcomes.^45^ Moreover, CKD patients on maintenance dialysis have been reported to be particularly vulnerable, with an increased risk of complications and mortality compared to the general population.^43^ Similarly, individuals with liver disease, such as cirrhosis or chronic liver disease, have also been shown to have an increased risk of severe COVID‐19 outcomes.^46^ Liver dysfunction, immune dysregulation, and impaired coagulation profiles in liver disease patients may contribute to the higher susceptibility and worse prognosis in this population.^47^, ^48^ These findings underscore the importance of recognizing CKD and liver disease as significant comorbidities in the context of COVID‐19 research and management.

Another limitation of this study is the heterogeneity among included studies. In the sensitivity analysis, the pooled estimates were calculated from studies with sample sizes larger than 1000. We were expecting that the exclusion of small studies may reduce the heterogeneity, but the contrary was observed. There were no large changes in either effect estimates or heterogeneity. In the network meta‐analysis, the heterogeneity was substantial when analyzed either by each outcome or the three outcomes combined. These differences between the included studies can be due to the heterogeneity of the methods, sample population, and model adjustments used by the individual studies identified. Differences in treatments,^49^, ^50^, ^51^ genetics, ethnicity,^52^ and location^13^, ^52^ may impact consistency of results. While heterogeneity was high in the conventional meta‐analysis, the network meta‐analysis can account for this, and our findings remain meaningful. The risk factor rankings of this network analysis allow for a more comprehensive comparison between cardiometabolic comorbidities. These findings provide a basis for clinical understanding of which conditions should be treated as greater risks.

This meta‐analysis and network analysis strengthened existing evidence on increased risk of COVID‐19 hospitalization, severity, and mortality among those with cardiometabolic comorbidities. The high prevalence and risk of cardiometabolic comorbidities should be accounted for when predicting prospects of the COVID‐19 pandemic.

## CONFLICT OF INTEREST STATEMENT

S.L. reports consulting payments and honoraria or promises of the same for scientific presentations or reviews at numerous venues, including but not limited to Barilla, Johns Hopkins University, Fred Hutchinson Cancer Center, Harvard University, University of Buffalo, Guangdong Provincial Hospital, Fuwai Hospital, the Chinese Academy of Medical Sciences, and the NIH. S.L. is also a member of the Data Safety and Monitoring Board for several trials, including the SELECT Trial‐Semaglutide Effects on Cardiovascular Outcomes in People with Overweight or Obesity sponsored by Novo Nordisk and a trial of pulmonary HTN in diabetes patients sponsored by Massachusetts General Hospital. S.L. receives royalties from UpToDate and receives an honorarium from the American Society for Nutrition for his duties as Associate Editor. Other authors report no competing interests.

## Supporting information


**Supplementary Table S1.** Search strategies.
**Supplementary Table S2.** Number of included studies in each group during the early and later pandemic.
**Supplementary Table S3.** Studies included in meta‐analysis (March 30, 2020 to May 29, 2021).
**Supplementary Figure S1.** Network graph of cardiometabolic risk factors comparisons from all included studies during COVID‐19 pandemic.
**Supplementary Figure S2.** Multiple comparison‐adjusted funnel plot of publication bias in relations of COVID‐19 composite outcome incidence in any cardiometabolic comorbidity.
**Supplementary Figure S3.** Network meta‐analysis on associations of cardiometabolic risk factors and COVID‐19 hospitalization.
**Supplementary Figure S4.** Network meta‐analysis on associations of cardiometabolic risk factors and COVID‐19 severity.
**Supplementary Figure S5.** Network meta‐analysis on associations of cardiometabolic risk factors and COVID‐19 mortality.
