# Supplementary material for: A network meta‐analysis of association between cardiometabolic risk factors and COVID‐19 outcome severity
Source: J Diabetes. 2023 Aug 30;15(11):968–77. doi: 10.1111/1753-0407.13445 (PMC10667650; doi:10.1111/1753-0407.13445)
Supplement: Supplementary file 1 — Supplementary Table S1. Search strategies. Supplementary Table S2. Number of included studies in each group during the early and later pandemic. Supplementary Table S3. Studies included in meta‐analysis (March 30, 2020 to May 29, 2021). Supplementary Figure S1. Network graph of cardiometabolic risk factors comparisons from all included studies during COVID‐19 pandemic. Supplementary Figure S2. Multiple comparison‐adjusted funnel plot of publication bias in relations of COVID‐19 composite outcome incidence in any cardiometabolic comorbidity. Supplementary Figure S3. Network meta‐analysis on associations of cardiometabolic risk factors and COVID‐19 hospitalization. Supplementary Figure S4. Network meta‐analysis on associations of cardiometabolic risk factors and COVID‐19 severity. Supplementary Figure S5. Network meta‐analysis on associations of cardiometabolic risk factors and COVID‐19 mortality. [file JDB-15-968-s001.docx]

**Supplementary Material**

Supplementary Table 1. Search strategies

Supplementary Table 2. Number of included studies in each group during the early and later pandemic

Supplementary Table 3. Studies Included in Meta-Analysis (March 30, 2020 to May 29, 2021).

Supplementary Figure 1. Network Graph of Cardiometabolic Risk Factors Comparisons from all Included Studies During COVID-19 Pandemic.

Supplementary Figure 2. Multiple Comparison-adjusted Funnel Plot of Publication Bias in Relations of COVID-19 composite outcome incidence in any cardiometabolic comorbidity.

Supplementary Figure 3. Network Meta-Analysis on Associations of Cardiometabolic Risk Factors and COVID-19 Hospitalization.

Supplementary Figure 4. Network Meta-Analysis on Associations of Cardiometabolic Risk Factors and COVID-19 Severity.

Supplementary Figure 5. Network Meta-Analysis on Associations of Cardiometabolic Risk Factors and COVID-19 Mortality.

**Supplementary Table 1**. Search strategies

| Databases |
| --- |
| Early pandemic:  1. Embase 1910 to May 19, 2021  2. Ovid Emcare 1995 to 2021 Week 19  3. Ovid MEDLINE(R) and Epub Ahead of Print, In-Process & Other Non-Indexed Citations 1946 to May 19, 2021 |
| Search terms |
| Exposure:  cardiometabolic OR cardiovascular disease OR CVD OR coronary heart disease OR CHD OR stroke OR cerebrovascular disease OR myocardial OR ischemic heart disease OR hyperten*  OR obes* OR overweight  OR diab* OR glucose intoleran* OR insulin resistance OR hyperglycem*  OR metabolic syndrome OR MetS OR Syndrome X OR insulin resistance syndrome OR dysmetabolic syndrome  OR dyslipidemia OR hyperlipidemia OR hypercholesterolemia OR hyperglyceridemia OR hyperlipoproteinemia  OR dyslipidaemia OR hyperlipidaemia OR hypercholesterolaemia OR hyperglyceridaemia OR hyperlipoproteinaemia  Study population:  severe acute respiratory syndrome coronavirus 2 OR 2019 ncov OR 2019nCoV OR sars cov 2 virus OR COVID-19 OR coronavirus disease 2019 virus OR SARS-CoV-2 OR SARS2 OR 2019-nCoV OR 2019 novel coronavirus |
| Inclusion criteria |
| 1. Primary studies with a cross-sectional, prospective, or retrospective design  2. All diagnosed patients with COVID-19  3. Studies that provided information on the association between cardiometabolic risk factors and outcomes related to COVID-19 (Hospitalization and length of stay; Disease severity as measured by days in ER, days in ICU, days on ventilator, and complications - blood clots (e.g., stroke, heart attacks, pulmonary embolism, deep vein thrombosis), kidney failure/dialysis, and Mortality)  4. Studies that provided effect estimates on the association between cardiometabolic risk factors and outcomes related to COVID-19, including odds ratio, relative risk, rate ratio, hazard ratio |
| Exclusion criteria |
| 1. Intervention studies, literature reviews, letters, or abstracts from conference proceedings  2. Studies that only reported prevalence of cardiometabolic comorbidities  3. Insufficient data information provided |

**Supplementary Table 2.** Number of included studies in each group

|  | Hospitalization | Severity | Mortality | Overall |
| --- | --- | --- | --- | --- |
| Atrial fibrillation | 4 | 2 | 7 | 13 |
| Coronary artery disease | 5 | 15 | 44 | 64 |
| Cerebrovascular disease | 3 | 15 | 22 | 40 |
| Cardiovascular disease | 12 | 32 | 51 | 95 |
| Diabetes mellitus | 32 | 125 | 177 | 334 |
| Heart failure | 11 | 16 | 47 | 74 |
| Hypertension | 22 | 97 | 135 | 254 |
| Myocardial infarction | 1 | 2 | 13 | 16 |
| Obesity | 21 | 67 | 79 | 167 |
| Overweight | 5 | 24 | 14 | 43 |
| Stroke | 4 | 7 | 16 | 27 |
| Overall | 120 | 402 | 605 | 1127 |

**Supplementary Table 3.** Studies Included in Meta-Analysis (March 30, 2020 to May 29, 2021).

| Author, year | Publication Title | NOS Score |
| --- | --- | --- |
| Abbas, 2020 | Presenting the characteristics, smoking versus diabetes, and outcome among patients hospitalized with COVID-19. | 7 |
| Abohamr, 2020 | Clinical characteristics and in-hospital mortality of covid-19 adult patients in saudi arabia. | 6 |
| Agarwal, 2020 | Cardiovascular Disease in Hospitalized Patients With a Diagnosis of Coronavirus From the Pre-COVID-19 Era in United States: National Analysis From 2016-2017. | 8 |
| Aghaaliakbari, 2020 | Angiotensin converting enzyme inhibitors, a risk factor of poor outcome in diabetic patients with COVID-19 infection. | 8 |
| Aidaoui, 2020 | Predictors of Severity in Covid-19 Patients in Casablanca, Morocco. | 9 |
| Al-Salameh, 2020 | Characteristics and outcomes of COVID-19 in hospitalized patients with and without diabetes. | 8 |
| Atkins, 2020 | Preexisting Comorbidities Predicting COVID-19 and Mortality in the UK Biobank Community Cohort. | 9 |
| Ayten, 2020 | Retrospective analysis of severe covid-19 pneumonia patients treated with lopinavir/ritonavir: A comparison with survivor and non-survivor patients. | 7 |
| Barbu, 2020 | The Impact of SARS-CoV-2 on the Most Common Comorbidities-A Retrospective Study on 814 COVID-19 Deaths in Romania. | 6 |
| Barman, 2020 | The effect of the severity COVID-19 infection on electrocardiography. | 7 |
| Barron, 2020 | Associations of type 1 and type 2 diabetes with COVID-19-related mortality in England: a whole-population study. | 9 |
| Bello-Chavolla, 2020 | Predicting Mortality Due to SARS-CoV-2: A Mechanistic Score Relating Obesity and Diabetes to COVID-19 Outcomes in Mexico. | 8 |
| Bello-Chavolla, 2020 | Unequal impact of structural health determinants and comorbidity on COVID-19 severity and lethality in older Mexican adults: Considerations beyond chronological aging. | 8 |
| Benussi, 2020 | Clinical characteristics and outcomes of inpatients with neurologic disease and COVID-19 in Brescia, Lombardy, Italy. | 7 |
| Bepouka, 2020 | Predictors of mortality in covid-19 patients at Kinshasa University Hospital, Democratic Republic of the Congo, from March to June 2020. | 9 |
| Bhargava, 2020 | Predictors for Severe COVID-19 Infection. | 5 |
| Bhatla, 2020 | COVID-19 and Cardiac Arrhythmias. | 7 |
| Burrell, 2020 | Outcomes for patients with COVID-19 admitted to Australian intensive care units during the first four months of the pandemic. | 8 |
| Busetto, 2020 | Obesity and COVID-19: an Italian snapshot. | 9 |
| Cai, 2020 | Association between obesity and clinical prognosis in patients infected with SARS-CoV-2. | 7 |
| Cai, 2020 | Obesity and COVID-19 Severity in a Designated Hospital in Shenzhen, China. | 9 |
| Cariou, 2020 | Phenotypic characteristics and prognosis of inpatients with COVID-19 and diabetes: the CORONADO study. | 8 |
| Carrillo-Vega, 2020 | Early estimation of the risk factors for hospitalization and mortality by COVID-19 in Mexico. | 8 |
| [Castelnuovo, 2020](https://www.sciencedirect.com/science/article/pii/S0939475320303069?casa_token=_ASbo4HWijEAAAAA:QpaAHOeB8CjlzW18sAjpCDbN60RgSecxMssQ-2bSo3gEBeuVxGR4o9miWzi3c0cogO4PxQzo3A#!) | Common cardiovascular risk factors and in-hospital mortality in 3,894 patients with COVID-19: survival analysis and machine learning-based findings from the multicentre Italian CORIST Study. | 9 |
| Cecconi, 2020 | Early predictors of clinical deterioration in a cohort of 239 patients hospitalized for Covid-19 infection in Lombardy, Italy. | 5 |
| Cen, 2020 | Risk factors for disease progression in COVID-19 patients. | 8 |
| Cen, 2020 | Risk factors for disease progression in patients with mild to moderate coronavirus disease 2019-a multi-centre observational study. | 8 |
| Chang, 2020 | COVID-19 Hospitalization by Race and Ethnicity: Association with Chronic Conditions Among Medicare Beneficiaries, January 1-September 30, 2020. | 9 |
| Chatrath, 2020 | The effect of concomitant COVID-19 infection on outcomes in patients hospitalized with heart failure. | 6 |
| Chen, 2020 | Coagulopathy is a major extrapulmonary risk factor for mortality in hospitalized patients with COVID-19 with type 2 diabetes. | 9 |
| Chen, 2020 | Clinical Characteristics and Outcomes of Patients With Diabetes and COVID-19 in Association With Glucose-Lowering Medication. | 6 |
| Chen, 2020 | The characteristics and outcomes of 681 severe cases with COVID-19 in China. | 7 |
| Chen, 2020 | Risk Factors of Fatal Outcome in Hospitalized Subjects With Coronavirus Disease 2019 From a Nationwide Analysis in China. | 7 |
| Cheng, 2020 | Clinical characteristics and fatal outcomes of hypertension in patients with severe COVID-19. | 8 |
| Chishinga, 2020 | Characteristics and Risk Factors for Hospitalization and Mortality among Persons with COVID-19 in Atlanta Metropolitan Area. | 8 |
| Chung, 2020 | The Risk of Diabetes on Clinical Outcomes in Patients with Coronavirus Disease 2019: A Retrospective Cohort Study. | 8 |
| Ciceri, 2020 | Early predictors of clinical outcomes of COVID-19 outbreak in Milan, Italy. | 6 |
| Claudia, 2020 | Characteristics, predictors and outcomes among 99 patients hospitalised with COVID-19 in a tertiary care centre in Switzerland: an observational analysis. | 6 |
| Cummings, 2020 | Epidemiology, clinical course, and outcomes of critically ill adults with COVID-19 in New York City: a prospective cohort study. | 8 |
| Deng, 2020 | Obesity as a Potential Predictor of Disease Severity in Young COVID-19 Patients: A Retrospective Study. | 6 |
| Deng, 2020 | The diagnostic and prognostic role of myocardial injury biomarkers in hospitalized patients with COVID-19. | 7 |
| [Denova-Gutiérrez, 2020](https://onlinelibrary.wiley.com/action/doSearch?ContribAuthorStored=Denova-Guti%C3%A9rrez%2C+Edgar) | The Association of Obesity, Type 2 Diabetes, and Hypertension with Severe Coronavirus Disease 2019 on Admission Among Mexican Patients. | 6 |
| Docherty, 2020 | Features of 20 133 UK patients in hospital with covid-19 using the ISARIC WHO Clinical Characterisation Protocol: Prospective observational cohort study. | 8 |
| Dong, 2020 | Development and Validation of a Nomogram for Assessing Survival in Patients with COVID-19 Pneumonia. | 5 |
| Eshrati, 2020 | Investigating the factors affecting the survival rate in patients with COVID-19: A retrospective cohort study. | 7 |
| Ferrante, 2020 | Risk factors for myocardial injury and death in patients with COVID-19: insights from a cohort study with chest computed tomography. | 8 |
| Galloway, 2020 | A clinical risk score to identify patients with COVID-19 at high risk of critical care admission or death: An observational cohort study. | 7 |
| Gao, 2020 | Association of hypertension and antihypertensive treatment with COVID-19 mortality: a retrospective observational study. | 7 |
| Garcia-Azorin, 2020 | Neurological Comorbidity Is a Predictor of Death in Covid-19 Disease: A Cohort Study on 576 Patients. | 8 |
| Giacomelli, 2020 | 30-day mortality in patients hospitalized with COVID-19 during the first wave of the Italian epidemic: A prospective cohort study. | 7 |
| Gobbato, 2020 | Clinical characteristics and risk factors associated with severe COVID-19: prospective analysis of 1,045 hospitalised cases in North-Eastern France, March 2020. | 7 |
| Golpe, 2020 | Risk of severe COVID-19 in hypertensive patients treated with renin-angiotensin-aldosterone system inhibitors. | 9 |
| Goodman, 2020 | Impact of Sex and Metabolic Comorbidities on COVID-19 Mortality Risk Across Age Groups: 66,646 Inpatients Across 613 U.S. Hospitals. | 8 |
| Görgülü, 2020 | Effects of Comorbid Factors on Prognosis of Three Different Geriatric Groups with COVID-19 Diagnosis. | 6 |
| Gottlieb, 2020 | Clinical Course and Factors Associated With Hospitalization and Critical Illness Among COVID-19 Patients in Chicago, Illinois. | 7 |
| Grasseli, 2020 | Risk Factors Associated With Mortality Among Patients With COVID-19 in Intensive Care Units in Lombardy, Italy. | 8 |
| Gu, 2020 | Characteristics Associated with Racial/Ethnic Disparities in COVID-19 Outcomes in an Academic Health Care System. | 9 |
| Guan, 2020 | Comorbidity and its impact on 1590 patients with COVID-19 in China: a nationwide analysis. | 8 |
| Hagg, 2020 | Age, Frailty, and Comorbidity as Prognostic Factors for Short-Term Outcomes in Patients With Coronavirus Disease 2019 in Geriatric Care. | 8 |
| Hajifathalian, 2020 | Obesity is associated with worse outcomes in COVID-19: Analysis of Early Data From New York City. | 6 |
| Hamer, 2020 | Lifestyle risk factors, inflammatory mechanisms, and COVID-19 hospitalization: A community-based cohort study of 387,109 adults in UK. | 6 |
| Hamer, 2020 | Lifestyle Risk Factors for Cardiovascular Disease in Relation to COVID-19 Hospitalization: A Community-Based Cohort Study of 387,109 Adults in UK. | 7 |
| Han, 2020 | Analysis of factors affecting the prognosis of COVID-19 patients and viral shedding duration. | 8 |
| Harmouch, 2020 | Is it all in the heart? Myocardial injury as major predictor of mortality among hospitalized COVID-19 patients. | 7 |
| Harrison, 2020 | Comorbidities associated with mortality in 31,461 adults with COVID-19 in the United States: A federated electronic medical record analysis. | 9 |
| Hashemi, 2020 | Impact of chronic liver disease on outcomes of hospitalized patients with COVID-19: A multicentre United States experience. | 8 |
| Heberto, 2020 | Implications of myocardial injury in Mexican hospitalized patients with coronavirus disease 2019 (COVID-19). | 6 |
| Hernández-Galdamez, 2020 | Increased Risk of Hospitalization and Death in Patients with COVID-19 and Pre-existing Noncommunicable Diseases and Modifiable Risk Factors in Mexico. | 9 |
| Hirsch, 2020 | Acute kidney injury in patients hospitalized with COVID-19. | 6 |
| Ho, 2020 | Modifiable and non-modifiable risk factors for COVID-19, and comparison to risk factors for influenza and pneumonia: Results from a UK Biobank prospective cohort study. | 7 |
| Hu, 2020 | Clinical epidemiological analyses of overweight/obesity and abnormal liver function contributing to prolonged hospitalization in patients infected with COVID-19. | 6 |
| Hu, 2020 | Risk Factors Associated with Clinical Outcomes in 323 COVID-19 Hospitalized Patients in Wuhan, China. | 6 |
| Hu, 2020 | Logistic regression analysis of death risk factors of patients with severe and critical coronavirus disease 2019 and their predictive value. [Chinese] | 7 |
| Hu, 2020 | Risk Factors Associated with Clinical Outcomes in 323 Coronavirus Disease 2019 (COVID-19) Hospitalized Patients in Wuhan, China. | 8 |
| Huang, 2020 | A novel risk score to predict cardiovascular complications in patients with coronavirus disease 2019 (COVID-19): A retrospective, multicenter, observational study. | 8 |
| Hultcrantz, 2020 | COVID-19 infections and outcomes in patients with multiple myeloma in New York City: a cohort study from five academic centers. | 3 |
| Hur, 2020 | Factors Associated With Intubation and Prolonged Intubation in Hospitalized Patients With COVID-19. | 9 |
| Hwang, 2020 | Neurological diseases as mortality predictive factors for patients with COVID-19: a retrospective cohort study. | 8 |
| Imam, 2020 | Older age and comorbidity are independent mortality predictors in a large cohort of 1305 COVID-19 patients in Michigan, United States. | 8 |
| Inciardi, 2020 | Characteristics and outcomes of patients hospitalized for COVID-19 and cardiac disease in Northern Italy. | 5 |
| Islam, 2020 | Risk factors associated with morbidity and mortality outcomes of COVID-19 patients on the 28th day of the disease course: A retrospective cohort study in Bangladesh. | 7 |
| Jackson, 2020 | Predictors at admission of mechanical ventilation and death in an observational cohort of adults hospitalized with COVID-19. | 8 |
| Jain, 2020 | A retrospective observational study to determine the early predictors of in-hospital mortality at admission with covid-19. | 7 |
| Jang, 2020 | Prognostic Factors for Severe Coronavirus Disease 2019 in Daegu, Korea. | 7 |
| Javanian, 2020 | Risk factors for mortality of 557 adult patients with COVID 19 in Babol, Northern Iran: a retrospective cohort study. | 7 |
| Jimeno, 2020 | Prognostic implications of neutrophil-lymphocyte ratio in COVID-19. | 5 |
| Kaeuffer, 2020 | Survival and predictors of deaths of patients hospitalised due to COVID-19 from a retrospective and multicentre cohort study in Brazil. | 7 |
| Kalligeros, 2020 | Association of Obesity with Disease Severity Among Patients with Coronavirus Disease 2019. | 6 |
| Kang, 2020 | Clinical characteristics of coronavirus disease 2019 patients with diarrhea in Daegu. | 8 |
| Kayem, 2020 | A snapshot of the Covid-19 pandemic among pregnant women in France. | 6 |
| Ken-Dror, 2020 | COVID-19 outcomes in UK centre within highest health and wealth band: A prospective cohort study. | 8 |
| Khawaja, 2020 | COVID-19 and its impact on the cardiovascular system. | 7 |
| Killerby, 2020 | Characteristics Associated with Hospitalization Among Patients with COVID-19 - Metropolitan Atlanta, Georgia, March-April 2020. | 5 |
| Kim, 2020 | The Correlation of Comorbidities on the Mortality in Patients with COVID-19: an Observational Study Based on the Korean National Health Insurance Big Data. | 8 |
| Kim, 2020 | Analysis of mortality and morbidity in covid-19 patients with obesity using clinical epidemiological data from the korean center for disease control & prevention. | 8 |
| Kim, 2020 | Clinical Characteristics and Outcomes of COVID-19 Cohort Patients in Daegu Metropolitan City Outbreak in 2020. | 8 |
| Kim, 2020 | Risk factors on the progression to clinical outcomes of covid-19 patients in south korea: Using national data. | 9 |
| Klang, 2020 | Morbid Obesity as an Independent Risk Factor for COVID-19 Mortality in Hospitalized Patients Younger than 50. | 8 |
| Kolhe, 2020 | Acute kidney injury associated with COVID-19: A retrospective cohort study. | 9 |
| Kuderer, 2020 | Clinical impact of COVID-19 on patients with cancer (CCC19): a cohort study. | 8 |
| Lala, 2020 | Prevalence and Impact of Myocardial Injury in Patients Hospitalized With COVID-19 Infection. | 9 |
| Lampasona, 2020 | Antibody response to multiple antigens of SARS-CoV-2 in patients with diabetes: an observational cohort study. | 7 |
| Lanini, 2020 | COVID-19 disease - Temporal analyses of complete blood count parameters over course of illness, and relationship to patient demographics and management outcomes in survivors and non-survivors: A longitudinal descriptive cohort study. | 9 |
| Lee, 2020 | Risk Factors for Mortality and Respiratory Support in Elderly Patients Hospitalized with COVID-19 in Korea. | 4 |
| Lee, 2020 | COVID-19 mortality in patients with cancer on chemotherapy or other anticancer treatments: a prospective cohort study. | 9 |
| Li, 2020 | Newly diagnosed diabetes is associated with a higher risk of mortality than known diabetes in hospitalized patients with COVID-19. | 5 |
| Li, 2020 | Baseline characteristics and risk factors for short-term outcomes in 132 COVID-19 patients with diabetes in Wuhan China: A retrospective study. | 6 |
| Li, 2020 | Clinical features and short-term outcomes of elderly patients with COVID-19. | 7 |
| Li, 2020 | Risk factors for severity and mortality in adult COVID-19 inpatients in Wuhan. | 8 |
| Lian, 2020 | High neutrophil-to-lymphocyte ratio associated with progression to critical illness in older patients with COVID-19: a multicenter retrospective study. | 8 |
| Liu, 2020 | Hyperglycemia is a strong predictor of poor prognosis in COVID-19. | 5 |
| Loannou, 2020 | Risk Factors for Hospitalization, Mechanical Ventilation, or Death among 10131 US Veterans with SARS-CoV-2 Infection. | 9 |
| Loffi, 2020 | Coronary artery disease in patients hospitalised with Coronavirus disease 2019 (COVID-19) infection. | 8 |
| Louapre, 2020 | Clinical Characteristics and Outcomes in Patients With Coronavirus Disease 2019 and Multiple Sclerosis. | 8 |
| Ma, 2020 | A nomogramic model based on clinical and laboratory parameters at admission for predicting the survival of COVID-19 patients. | 7 |
| Maddaloni, 2020 | Cardiometabolic multimorbidity is associated with a worse Covid-19 prognosis than individual cardiometabolic risk factors: A multicentre retrospective study (CoViDiab II). | 7 |
| Mccullough, 2020 | Electrocardiographic Findings in Coronavirus Disease-19: Insights on Mortality and Underlying Myocardial Processes. | 7 |
| Mallow, 2020 | Outcomes of hospitalized COVID-19 patients by risk factors: Results from a United States hospital claims database. | 7 |
| Marcedo, 2020 | Correlation between hospitalized patients' demographics, symptoms, comorbidities, and COVID-19 pandemic in Bahia, Brazil. | 5 |
| Martins-Filho, 2020 | Factors associated with mortality among hospitalized patients with covid-19: A retrospective cohort study. | 6 |
| McCarty, 2020 | How Do Presenting Symptoms and Outcomes Differ by Race/Ethnicity Among Hospitalized Patients with COVID-19 Infection? Experience in Massachusetts. | 9 |
| Mehra, 2020 | Cardiovascular disease, drug therapy, and mortality in COVID-19. | 7 |
| Mehra, 2020 | Hydroxychloroquine or chloroquine with or without a macrolide for treatment of COVID-19: a multinational registry analysis. | 7 |
| Mendy, 2020 | Factors Associated with Hospitalization and Disease Severity in a Racially and Ethnically Diverse Population of COVID-19 Patients. | 8 |
| Mirani, 2020 | Impact of comorbidities and glycemia at admission and dipeptidyl peptidase 4 inhibitors in patients with type 2 diabetes with covid-19: A case series from an academic hospital in lombardy, italy. | 8 |
| Moon, 2020 | Independent impact of diabetes on the severity of coronavirus disease 2019 in 5,307 patients in South Korea: A nationwide cohort study. | 8 |
| Munblit, 2020 | StopCOVID cohort: An observational study of 3,480 patients admitted to the Sechenov University hospital network in Moscow city for suspected COVID-19 infection. | 9 |
| Nakeshbandi, 2020 | The impact of obesity on COVID-19 complications: a retrospective cohort study. | 8 |
| Nie, 2020 | Epidemiological and clinical characteristics of 671 COVID-19 patients in Henan Province, China. | 8 |
| Nimkar, 2020 | Incidence and Risk Factors for Acute Kidney Injury and Its Effect on Mortality in Patients Hospitalized From COVID-19. | 9 |
| Núñez-Gil, 2020 | Underlying heart diseases and acute COVID-19 outcomes. | 9 |
| Oetjens, 2020 | Electronic health record analysis identifies kidney disease as the leading risk factor for hospitalization in confirmed COVID-19 patients. | 9 |
| Olivas-Martinez A., 2020 | In-hospital mortality from severe COVID-19 in a tertiary care center in Mexico City; causes of death, risk factors and the impact of hospital saturation. | 9 |
| Omrani, 2020 | The first consecutive 5000 patients with Coronavirus Disease 2019 from Qatar; a nation-wide cohort study. | 8 |
| Palaiodimos, 2020 | Severe obesity, increasing age and male sex are independently associated with worse in-hospital outcomes, and higher in-hospital mortality, in a cohort of patients with COVID-19 in the Bronx, New York. | 7 |
| Palaiodimos, 2020 | Severe obesity is associated with higher in-hospital mortality in a cohort of patients with COVID-19 in the Bronx, New York. | 8 |
| Pan, 2020 | Clinical Features of COVID-19 in Patients With Essential Hypertension and the Impacts of Renin-angiotensin-aldosterone System Inhibitors on the Prognosis of COVID-19 Patients. | 6 |
| Park, 2020 | Impact of Cardiovascular Risk Factors and Cardiovascular Diseases on Outcomes in Patients Hospitalized with COVID-19 in Daegu Metropolitan City. | 5 |
| Peng, 2020 | Clinical characteristics and prognosis of 244 cardiovascular patients suffering from coronavirus disease in Wuhan, China. | 8 |
| Perez, 2020 | Comorbidity and prognostic factors on admission in a COVID-19 cohort of a general hospital. | 6 |
| Pettit, 2020 | Obesity is Associated with Increased Risk for Mortality Among Hospitalized Patients with COVID-19. | 7 |
| Phipps, 2020 | Acute Liver Injury in COVID-19: Prevalence and Association with Clinical Outcomes in a Large US Cohort. | 8 |
| Poblador-Plou, 2020 | Baseline chronic comorbidity and mortality in laboratory-confirmed COVID-19 cases: Results from the PRECOVID study in Spain. | 9 |
| Polverino, 2020 | Comorbidities, cardiovascular therapies, and COVID-19 mortality: A nationwide, italian observational study (ItaliCO). | 7 |
| Price-Haywood, 2020 | Hospitalization and mortality among black patients and white patients with Covid-19. | 8 |
| Rastad, 2020 | Risk and predictors of in-hospital mortality from COVID-19 in patients with diabetes and cardiovascular disease. | 9 |
| Redondo-Bravo, 2020 | The first wave of the COVID-19 pandemic in Spain: Characterisation of cases and risk factors for severe outcomes, as at 27 April 2020. | 7 |
| Reilev, 2020 | Characteristics and predictors of hospitalization and death in the first 11 122 cases with a positive RT-PCR test for SARS-CoV-2 in Denmark: A nationwide cohort. | 9 |
| Romagnolo, 2020 | Neurological comorbidity and severity of COVID-19. | 8 |
| Rossi, 2020 | Characteristics and outcomes of a cohort of COVID-19 patients in the Province of Reggio Emilia, Italy. | 9 |
| Rottoli, 2020 | How important is obesity as a risk factor for respiratory failure, intensive care admission and death in hospitalised COVID-19 patients? Results from a single Italian centre. | 7 |
| Rozaliyani, 2020 | Factors Associated with Death in COVID-19 Patients in Jakarta, Indonesia: An Epidemiological Study. | 9 |
| Sabri, 2020 | Novel coronavirus disease 2019: predicting prognosis by using a computed tomography severity score and clinicolaboratory data. | 6 |
| Salacup, 2020 | Characteristics and clinical outcomes of COVID-19 patients in an underserved-inner city population: A single tertiary center cohort. | 7 |
| Saleh, 2020 | The association between cardiac injury and outcomes in hospitalized patients with COVID-19. | 9 |
| Sands, 2020 | Patient characteristics and admitting vital signs associated with coronavirus disease 2019 (COVID-19)-related mortality among patients admitted with noncritical illness. | 7 |
| Santos, 2020 | Determinants of COVID-19 disease severity in patients with underlying rheumatic disease. | 9 |
| Santos, 2020 | Clinical, demographical characteristics and hospitalisation of 3,010 patients with Covid-19 in Friuli Venezia Giulia Region (Northern Italy). A multivariate, population-based, statistical analysis. | 7 |
| Seiglie, 2020 | Diabetes as a risk factor for poor early outcomes in patients hospitalized with covid-19. | 8 |
| Shah, 2020 | Demographics, comorbidities and outcomes in hospitalized Covid-19 patients in rural southwest Georgia. | 8 |
| Shah, 2020 | Factors associated with increased mortality in hospitalized COVID-19 patients. | 8 |
| Shang, 2020 | The Relationship between Diabetes Mellitus and COVID-19 Prognosis: A Retrospective Cohort Study in Wuhan, China. | 8 |
| Shi, 2020 | Association of Cardiac Injury with Mortality in Hospitalized Patients with COVID-19 in Wuhan, China. | 8 |
| Simonnet, 2020 | High Prevalence of Obesity in Severe Acute Respiratory Syndrome Coronavirus-2 (SARS-CoV-2) Requiring Invasive Mechanical Ventilation. | 8 |
| Soares, 2020 | Risk Factors for Hospitalization and Mortality due to COVID-19 in Espirito Santo State, Brazil. | 9 |
| Sousa, 2020 | Mortality and survival of COVID-19. | 5 |
| Suleyman, 2020 | Clinical Characteristics and Morbidity Associated with Coronavirus Disease 2019 in a Series of Patients in Metropolitan Detroit. | 8 |
| Sun, 2020 | Independent and combined effects of hypertension and diabetes on clinical outcomes in patients with COVID-19: A retrospective cohort study of Huoshen Mountain Hospital and Guanggu Fangcang Shelter Hospital. | 8 |
| Sutter, 2020 | Association of diabetes and outcomes in patients with COVID-19: Propensity score-matched analyses from a French retrospective cohort. | 8 |
| Thompson, 2020 | Patient characteristics and predictors of mortality in 470 adults admitted to a district general hospital in England with Covid-19. | 9 |
| Toussie, 2020 | Clinical and Chest Radiography Features Determine Patient Outcomes In Young and Middle Age Adults with COVID-19. | 5 |
| Turcotte, 2020 | Risk factors for severe illness in hospitalized Covid-19 patients at a regional hospital. | 8 |
| Vena, 2020 | Clinical characteristics, management and in-hospital mortality of patients with coronavirus disease 2019 in Genoa, Italy. | 8 |
| Wang, 2020 | Coronavirus disease 2019 in elderly patients: Characteristics and prognostic factors based on 4-week follow-up. | 7 |
| Wang, 2020 | Fasting blood glucose at admission is an independent predictor for 28-day mortality in patients with COVID-19 without previous diagnosis of diabetes: a multi-centre retrospective study. | 9 |
| Wang, 2020 | Clinical Features of COVID-19 Patients with Different Outcomes in Wuhan: A Retrospective Observational Study. | 4 |
| Wang, 2020 | Overweight and Obesity are Risk Factors of Severe Illness in Patients with COVID-19. | 8 |
| Wang, 2020 | Epidemiological characteristics of patients with severe COVID-19 infection in Wuhan, China: evidence from a retrospective observational study. | 9 |
| Watanabe, 2020 | Visceral fat shows the strongest association with the need of intensive Care in Patients with COVID-19. | 7 |
| Wei, 2020 | Acute myocardial injury is common in patients with COVID-19 and impairs their prognosis. | 8 |
| Williamson, 2020 | OpenSAFELY: factors associated with COVID-19 death in 17 million patients. | 4 |
| Wollenstein-Betech, 2020 | Physiological and socioeconomic characteristics predict COVID-19 mortality and resource utilization in Brazil. | 7 |
| Wu, 2020 | Risk Factors Associated with Acute Respiratory Distress Syndrome and Death in Patients with Coronavirus Disease 2019 Pneumonia in Wuhan, China. | 5 |
| Wu, 2020 | Early antiviral treatment contributes to alleviate the severity and improve the prognosis of patients with novel coronavirus disease (COVID-19). | 5 |
| Wu, 2020 | Influence of diabetes mellitus on the severity and fatality of SARS-CoV-2 (COVID-19) infection. | 5 |
| Wu, 2020 | Influence of diabetes mellitus on the severity and fatality of SARS-CoV-2 infection. | 7 |
| Xiao, 2020 | Development and validation of the HNC-LL score for predicting the severity of coronavirus disease 2019. | 8 |
| Xie, 2020 | Association Between Hypoxemia and Mortality in Patients With COVID-19. | 5 |
| Xiong, 2020 | Hypertension is a risk factor for adverse outcomes in patients with coronavirus disease 2019: a cohort study. | 7 |
| Xu, 2020 | Factors associated with prolonged viral RNA shedding in patients with COVID-19. | 8 |
| Xu, 2020 | Factors Associated With Prolonged Viral RNA Shedding in Patients with Coronavirus Disease 2019 (COVID-19). | 8 |
| Xu, 2020 | Diabetic patients with COVID-19 need more attention and better glycemic control. | 8 |
| Yan, 2020 | Clinical characteristics and outcomes of patients with severe covid-19 with diabetes. | 8 |
| Yan, 2020 | Association of Overlapped and Un-overlapped Comorbidities with COVID-19 Severity and Treatment Outcomes: A Retrospective Cohort Study from Nine Provinces in China. | 8 |
| Yang, 2020 | Effect of hypertension on outcomes of adult inpatients with COVID-19 in Wuhan, China: a propensity score-matching analysis. | 6 |
| Yang, 2020 | Risks factors for death among COVID-19 patients combined with hypertension, coronary heart disease or diabetes. [Chinese] | 7 |
| Yang, 2020 | Visceral Adiposity and High Intramuscular Fat Deposition Independently Predict Critical Illness in Patients with Sars-COV-2. | 8 |
| Yazdanpanah, 2020 | Impact on disease mortality of clinical, biological, and virological characteristics at hospital admission and overtime in COVID-19 patients. | 8 |
| Ye, 2020 | Impact of comorbidities on patients with COVID-19: A large retrospective study in Zhejiang, China. | 8 |
| Yip, 2020 | Liver injury is independently associated with adverse clinical outcomes in patients with COVID-19. | 7 |
| You, 2020 | Clinical outcomes of COVID-19 patients with type 2 diabetes: A population-based study in Korea. | 8 |
| Yu, 2020 | Clinical Characteristics, Associated Factors, and Predicting COVID-19 Mortality Risk: A Retrospective Study in Wuhan, China. | 7 |
| Yun, 2020 | Severe COVID-19 Illness: Risk Factors and Its Burden on Critical Care Resources. | 7 |
| Zamanian, 2020 | Association of underlying diseases and clinical characteristics with mortality in patients with 2019 novel coronavirus in Iran. | 5 |
| Zandkarimi, 2020 | The Prognostic Factors Affecting the Survival of Kurdistan Province COVID-19 Patients: A Cross-sectional Study From February to May 2020. | 7 |
| Zeng, 2020 | Simple nomogram based on initial laboratory data for predicting the probability of ICU transfer of COVID-19 patients: Multicenter retrospective study. | 5 |
| Zeuschner, 2020 | Should We Perform Old-for-Old Kidney Transplantation during the COVID-19 Pandemic? The Risk for Post-Operative Intensive Stay. | 4 |
| Zhang, 2020 | Association of diabetes mellitus with disease severity and prognosis in COVID-19: A retrospective cohort study. | 5 |
| Zhang, 2020 | Do underlying cardiovascular diseases have any impact on hospitalised patients with COVID-19?. | 7 |
| Zhang, 2020 | Clinical outcomes of COVID-19 cases and influencing factors in Guangdong province. [Chinese] | 8 |
| Zhang, 2020 | Clinical Course and Mortality of Stroke Patients with Coronavirus Disease 2019 in Wuhan, China. | 9 |
| Zhao, 2020 | Comparison of clinical characteristics and outcomes of patients with coronavirus disease 2019 at different ages. | 6 |
| Zhou, 2020 | Development and validation a nomogram for predicting the risk of severe COVID-19: A multi-center study in Sichuan, China. | 5 |
| Zhou, 2020 | Clinical course and risk factors for mortality of adult inpatients with COVID-19 in Wuhan, China: a retrospective cohort study. | 8 |
| Zhu, 2020 | Association of obesity and its genetic predisposition with the risk of severe COVID-19: Analysis of population-based cohort data. | 7 |
| Zhu, 2020 | Clinical value of immune-inflammatory parameters to assess the severity of coronavirus disease 2019. | 7 |
| Abraha, 2021 | Clinical features and risk factors associated with morbidity and mortality among patients with COVID-19 in northern Ethiopia. | 8 |
| Andreano, 2021 | Development of a multivariable model predicting mortality risk from comorbidities in an Italian cohort of 18,286 confirmed COVID-19 cases aged 40 years or older. | 9 |
| Aslaner, 2021 | The effect of chronic diseases, age and gender on morbidity and mortality of covid-19 infection. | 8 |
| Bergman, 2021 | Risk factors for COVID-19 diagnosis, hospitalization, and subsequent all-cause mortality in Sweden: a nationwide study. | 9 |
| Bhatt, 2021 | Clinical Outcomes in Patients With Heart Failure Hospitalized With COVID-19. | 9 |
| Bonifazi, 2021 | Predictors of worse prognosis in young and middle-aged adults hospitalized with covid-19 pneumonia: A multi-center italian study (covid-under50). | 9 |
| Byeon, 2021 | Factors affecting the survival of early COVID-19 patients in South Korea: An observational study based on the Korean National Health Insurance big data. | 7 |
| Cao, 2021 | Obesity and COVID-19 in Adult Patients With Diabetes. | 7 |
| Cervantes, 2021 | Factors associated with COVID-19 severity and mortality among Hispanic patients living on the USA-Mexico border. | 8 |
| Chan, 2021 | COVID-19 in the New York City Jail System: Epidemiology and Health Care Response, March-April 2020. | 9 |
| Chetboun, 2021 | BMI and pneumonia outcomes in critically ill COVID-19 patients: an international multicenter study. | 9 |
| Cho, 2021 | Impact of comorbidity burden on mortality in patients with COVID-19 using the Korean health insurance database. | 7 |
| Ciardullo, 2021 | Impact of diabetes on COVID-19-related in-hospital mortality: a retrospective study from Northern Italy. | 8 |
| Cottini, 2021 | Obesity is a Major Risk Factor for Hospitalization in Community-Managed COVID-19 Pneumonia. | 9 |
| Crouse, 2021 | Metformin Use Is Associated With Reduced Mortality in a Diverse Population With COVID-19 and Diabetes. | 7 |
| Cummins, 2021 | Factors associated with COVID-19 related hospitalisation, critical care admission and mortality using linked primary and secondary care data. | 9 |
| Dennis, 2021 | Type 2 diabetes and covid-19- related mortality in the critical care setting: A national cohort study in england, march-july 2020. | 8 |
| Desai, 2021 | The role of anti-hypertensive treatment, comorbidities and early introduction of LMWH in the setting of COVID-19: A retrospective, observational study in Northern Italy. | 8 |
| El-Jawahri, 2021 | Clinical Outcomes of Patients Hospitalized with Coronavirus Disease 2019 (COVID-19) in Boston. | 8 |
| Esme, 2021 | Older Adults With Coronavirus Disease 2019: A Nationwide Study in Turkey. | 9 |
| Fang, 2021 | Impact of comorbidities on clinical prognosis in 1280 patients with different types of COVID-19. | 8 |
| Fresán, 2021 | Hypertension and related comorbidities as potential risk factors for covid-19 hospitalization and severity: A prospective population-based cohort study. | 9 |
| Gao, 2021 | Associations between body-mass index and COVID-19 severity in 6.9 million people in England: a prospective, community-based, cohort study. | 9 |
| Geng, 2021 | Risk factors for developing severe COVID-19 in China: an analysis of disease surveillance data. | 8 |
| Geteneh, 2021 | Clinical characteristics of patients infected with sars-cov-2 in north wollo zone, north-east ethiopia. | 7 |
| Girardin, 2021 | Contribution of pulmonary diseases to COVID-19 mortality in a diverse urban community of New York. | 8 |
| Gray, 2021 | Changes in COVID-19 in-hospital mortality in hospitalised adults in England over the first seven months of the pandemic: An observational study using administrative data. | 9 |
| Gregory, 2021 | COVID-19 severity is tripled in the diabetes community: A prospective analysis of the pandemic's impact in type 1 and type 2 diabetes. | 8 |
| Gude-Sampedro, 2021 | Development and validation of a prognostic model based on comorbidities to predict COVID-19 severity: a population-based study. | 7 |
| Gupta, 2021 | Diabetes Mellitus and Hypertension Increase Risk of Death in Novel Corona Virus Patients Irrespective of Age: a Prospective Observational Study of Co-morbidities and COVID-19 from India. | 6 |
| He, 2021 | The Prognostic Value of Myocardial Injury in COVID-19 Patients and Associated Characteristics. | 8 |
| Henein, 2021 | Combined cardiac risk factors predict covid-19 related mortality and the need for mechanical ventilation in coptic clergy. | 8 |
| Hobbs, 2021 | Risk factors for mortality and progression to severe covid-19 disease in the Southeast United States (US): A report from the SEUS study Group. | 8 |
| Ilic, 2021 | Pneumonia in healthcare workers during a COVID-19 outbreak at a cardiovascular hospitals. | 7 |
| Ioannou, 2021 | Development of COVIDVax Model to Estimate the Risk of SARS-CoV-2-Related Death Among 7.6 Million US Veterans for Use in Vaccination Prioritization. | 8 |
| Jakob, 2021 | First results of the "Lean European Open Survey on SARS-CoV-2-Infected Patients (LEOSS)". | 8 |
| Jaspard, 2021 | Clinical presentation, outcomes and factors associated with mortality: a prospective study from three COVID-19 referral care centers in West Africa. | 7 |
| Jayanama, 2021 | The association between body mass index and severity of Coronavirus Disease 2019 (COVID-19): A cohort study. | 4 |
| Keski, 2021 | Hematological and Inflammatory Parameters to Predict the Prognosis in COVID-19. | 8 |
| Khan, 2021 | Clinical characteristics and outcomes of patients with Corona Virus Disease 2019 (COVID-19) at Mercy Health Hospitals, Toledo, Ohio. | 9 |
| Kim, 2021 | Evaluation of the prognosis of covid-19 patients according to the presence of underlying diseases and drug treatment. | 8 |
| Kim, 2021 | Risk Factors for Intensive Care Unit Admission and In-hospital Mortality Among Hospitalized Adults Identified through the US Coronavirus Disease 2019 (COVID-19)-Associated Hospitalization Surveillance Network (COVID-NET). | 9 |
| Koh, 2021 | Diabetes predicts severity of COVID-19 infection in a retrospective cohort: A mediatory role of the inflammatory biomarker C-reactive protein. | 8 |
| Laake, 2021 | Characteristics, management and survival of ICU patients with coronavirus disease-19 in Norway, March-June 2020. A prospective observational study. | 7 |
| León-Pedroza, 2021 | Impact of metabolic syndrome in the clinical outcome of disease by SARS-COV-2 in Mexican population. | 6 |
| Liu, 2021 | Diabetes, even newly defined by HbA1c testing, is associated with an increased risk of in-hospital death in adults with COVID-19. | 9 |
| Liu, 2021 | Clinical characteristics and related risk factors of disease severity in 101 COVID-19 patients hospitalized in Wuhan, China. | 7 |
| Lucar, 2021 | Epidemiology, Clinical Features, and Outcomes of Hospitalized Adults with COVID-19: Early Experience from an Academic Medical Center in Mississippi. | 8 |
| Magro, 2021 | Predicting in-hospital mortality from Coronavirus Disease 2019: A simple validated app for clinical use. | 8 |
| Manohar, 2021 | Social and Clinical Determinants of COVID-19 Outcomes: Modeling Real-World Data from a Pandemic Epicenter. | 7 |
| Marcolino, 2021 | Clinical characteristics and outcomes of patients hospitalized with COVID-19 in Brazil: Results from the Brazilian COVID-19 registry. | 8 |
| Mbarga, 2021 | Clinical profile and factors associated with COVID-19 in Yaounde, Cameroon: A prospective cohort study. | 7 |
| Mendes, 2021 | Incidence, characteristics and clinical relevance of acute stroke in old patients hospitalized with COVID-19. | 4 |
| Morys, 2021 | Poor Metabolic Health Increases COVID-19-Related Mortality in the UK Biobank Sample. | 8 |
| Motaib, 2021 | Obesity and Disease Severity Among Patients With COVID-19. | 9 |
| Najera, 2021 | Health and Institutional Risk Factors of COVID-19 Mortality in Mexico, 2020. | 8 |
| Nascimento, 2021 | COVID-19 and Myocardial Injury in a Brazilian ICU: High Incidence and Higher Risk of In-Hospital Mortality. [Portuguese, English] | 7 |
| Ngiam, 2021 | Demographic shift in COVID-19 patients in Singapore from an aged, at-risk population to young migrant workers with reduced risk of severe disease. | 9 |
| Nijman, 2021 | Risk factors for in-hospital mortality in laboratory-confirmed COVID-19 patients in the Netherlands: A competing risk survival analysis. | 8 |
| Niu, 2021 | Development of a predictive model for mortality in hospitalized patients with COVID-19. | 7 |
| Osibogun, 2021 | Outcomes of COVID-19 patients with comorbidities in southwest Nigeria. | 7 |
| Panagiotou, 2021 | Risk Factors Associated with All-Cause 30-Day Mortality in Nursing Home Residents with COVID-19. | 8 |
| Paranjape, 2021 | Development and validation of a predictive model for critical illness in adult patients requiring hospitalization for COVID-19. | 9 |
| Peterson, 2021 | The relationship between coronary artery disease and clinical outcomes in COVID-19: a single-center retrospective analysis. | 6 |
| Pettrone, 2021 | Characteristics and risk factors of hospitalized and nonhospitalized COVID-19 patients, Atlanta, Georgia, USA, March-April 2020. | 8 |
| Pishgahi, 2021 | Echocardiographic Abnormalities as Independent Prognostic Factors of In-Hospital Mortality among COVID-19 Patients. | 5 |
| Ricchio, 2021 | Characteristics, Management, and Outcomes of Elderly Patients with Diabetes in a Covid-19 Unit: Lessons Learned from a Pilot Study. | 5 |
| Rodriguez-Gonzalez, 2021 | COVID-19 in hospitalised patients in Spain: a cohort study in Madrid. | 6 |
| Rossi, 2021 | Obesity as a risk factor for unfavourable outcomes in critically ill patients affected by Covid 19. | 7 |
| Rumery, 2021 | Outcomes of coronavirus disease-2019 among veterans with pre-existing diagnosis of heart failure. | 7 |
| Satman, 2021 | Unexpectedly lower mortality rates in COVID-19 patients with and without type 2 diabetes in Istanbul. | 8 |
| Sharif, 2021 | Prevalence and impact of diabetes and cardiovascular disease on clinical outcome among patients with COVID-19 in Bangladesh. | 7 |
| Shin, 2021 | Anatomy of comorbidity complexity of COVID-19: the case of hospitalized South Korean patients. | 9 |
| Shrestha, 2021 | Type 2 diabetes is associated with increased risk of critical respiratory illness in patients COVID-19 in a community hospital. | 5 |
| Silva, 2021 | Risk factors for critical illness and death among adult Brazilians with COVID-19. | 7 |
| Silverii, 2021 | Are diabetes and its medications risk factors for the development of COVID-19? Data from a population-based study in Sicily. | 5 |
| Sonmez, 2021 | Clinical characteristics and outcomes of COVID-19 in patients with type 2 diabetes in Turkey: A nationwide study (TurCoviDia). | 7 |
| Souza, 2021 | Clinical course and outcome of patients with COVID-19 in Mumbai City: An observational study. | 6 |
| Surendra, 2021 | Clinical characteristics and mortality associated with COVID-19 in Jakarta, Indonesia: A hospital-based retrospective cohort study. | 9 |
| Suresh, 2021 | Association of obesity with illness severity in hospitalized patients with COVID-19: A retrospective cohort study. | 8 |
| Tang, 2021 | A comprehensive evaluation of early potential risk factors for disease aggravation in patients with COVID-19. | 8 |
| Tchang, 2021 | The Independent Risk of Obesity and Diabetes and Their Interaction in COVID-19: A Retrospective Cohort Study. | 9 |
| Terlecki, 2021 | Association between cardiovascular disease, cardiovascular drug therapy, and in-hospital outcomes in patients with COVID-19: data from a large single-center registry in Poland. | 8 |
| Tessitore, 2021 | Mortality and high risk of major adverse events in patients with COVID-19 and history of cardiovascular disease. | 8 |
| Toh, 2021 | Outcomes for the first wave of hospitalised patients with COVID-19 in the South Australian context: a retrospective audit. | 5 |
| Toprak, 2021 | Do age, hypertension, coronary artery disease, ace-i, ARB or beta-blockers therapy increase the risk of mortality in COVID 19 patients? The results of a tertiary center in Turkey. | 5 |
| Tsai, 2021 | COVID-19 associated mortality and cardiovascular disease outcomes among US women veterans. | 8 |
| Vaughan, 2021 | Relationship of socio-demographics, comorbidities, symptoms and healthcare access with early COVID-19 presentation and disease severity. | 8 |
| Vera-Zertuche, 2021 | Obesity is a strong risk factor for short-term mortality and adverse outcomes in Mexican patients with COVID-19: A national observational study. | 7 |
| Vergara, 2021 | Role of comorbidities on the mortality in patients with SARS-CoV-2 infection: an Italian cohort study. | 8 |
| Villalba, 2021 | Impact of the presence of heart disease, cardiovascular medications and cardiac events on outcome in COVID-19. | 7 |
| Woolcott, 2021 | The effect of age on the association between diabetes and mortality in adult patients with COVID-19 in Mexico. | 9 |
| Yoshida, 2021 | Clinical characteristics and outcomes in women and men hospitalized for coronavirus disease 2019 in New Orleans. | 8 |
| Zhang, 2021 | The association between obesity and severity in patients with coronavirus disease 2019: A retrospective, single-center study, Wuhan. | 9 |
| Zhang, 2021 | Risk factors associated with the progression of COVID-19 in elderly diabetes patients. | 6 |
| Zhong R., 2021 | Which Factors, Smoking, Drinking Alcohol, Betel Quid Chewing, or Underlying Diseases, Are More Likely to Influence the Severity of COVID-19?. | 6 |

**Supplementary** **Figure 1.** Network Graph of Cardiometabolic Risk Factors Comparisons from all Included Studies During COVID-19 Pandemic.


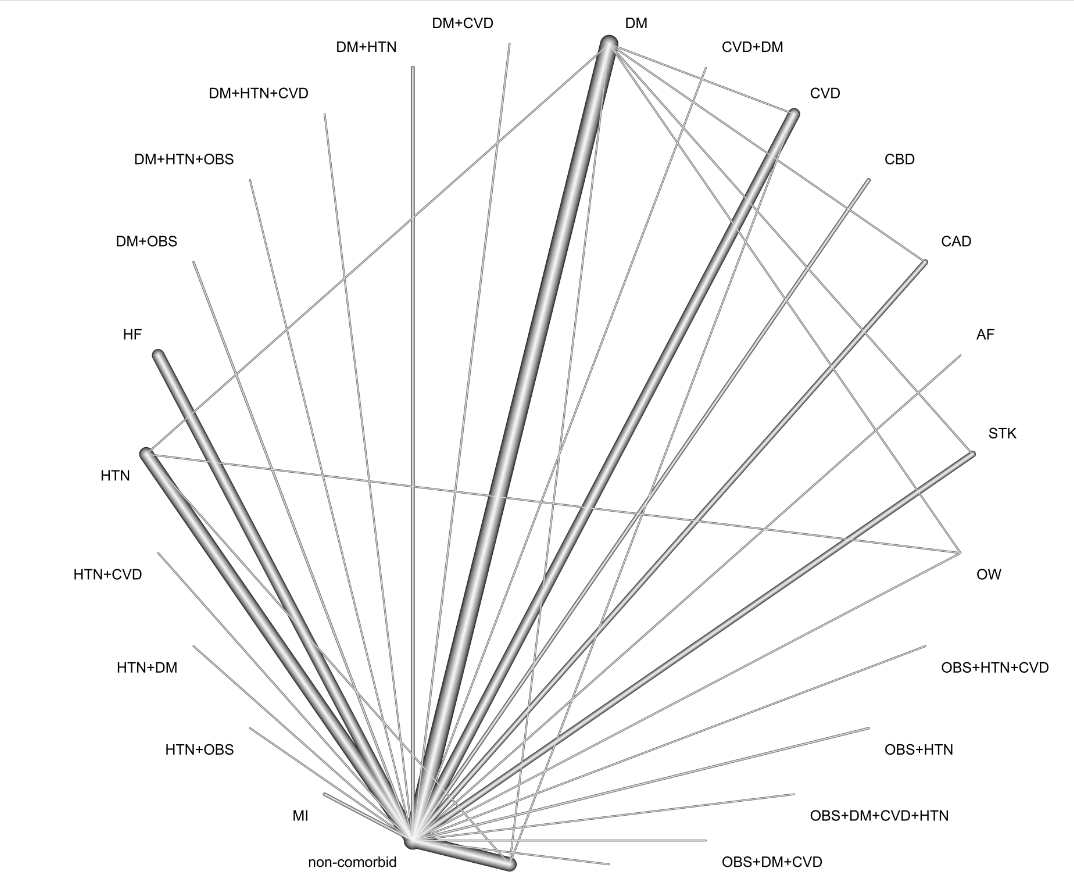


Abbreviations: AF, atrial fibrillation; CAD, coronary artery disease; CBD, cerebrovascular disease; CHD, coronary heart disease; CVD, cardiovascular disease; DM, diabetes mellitus; HF, heart failure; HTN, hypertension; MI, myocardial infarction; OBS, obesity; OW, overweight; STK, stroke.

Cardiometabolic factors were compared to a non-comorbid category of having none of the cardiometabolic risk factors studied.

**Supplementary Figure 2**. Multiple Comparison-adjusted Funnel Plot of Publication Bias in Relations of COVID-19 composite outcome incidence in any cardiometabolic comorbidity.


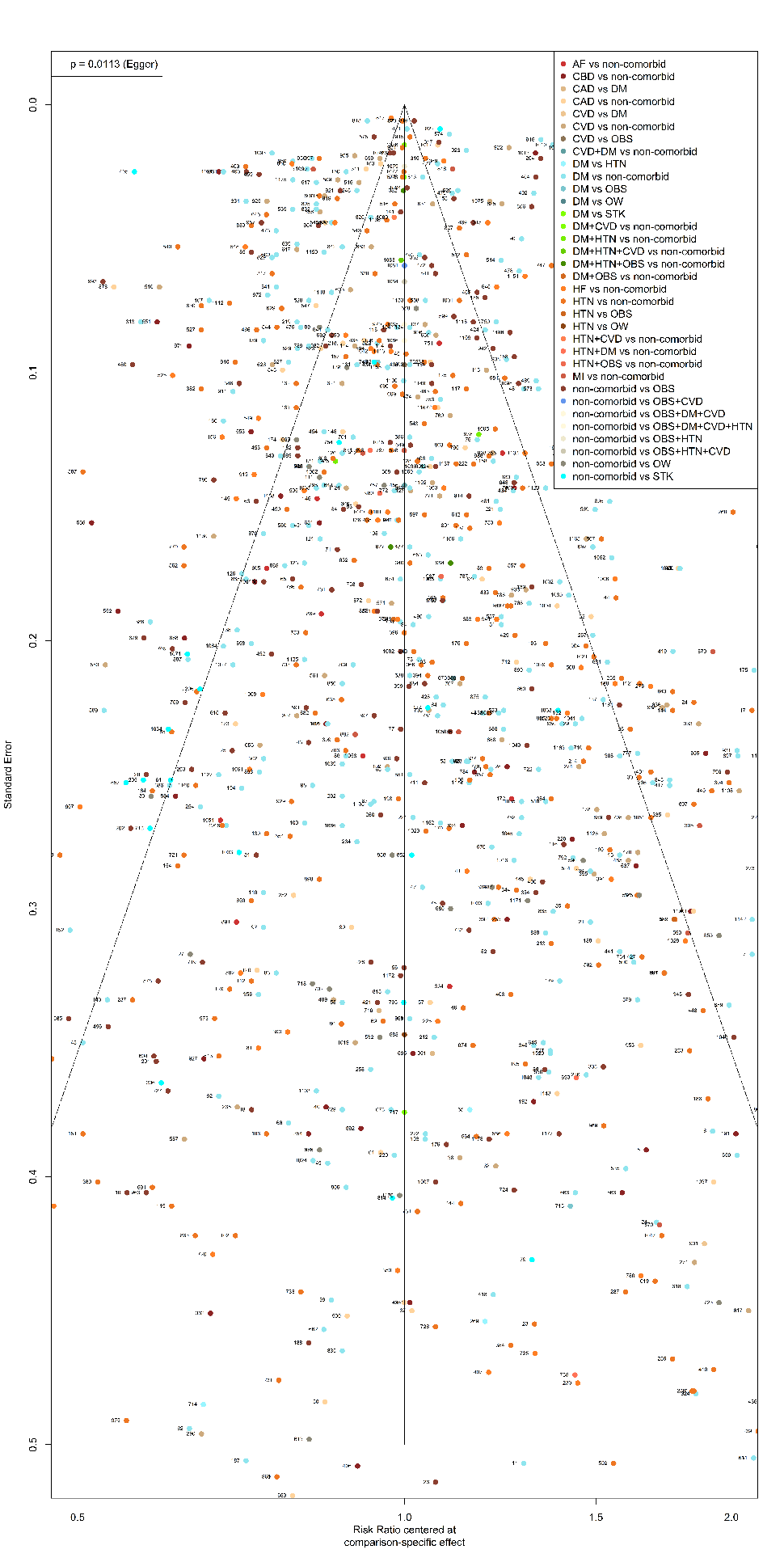


**Supplementary Figure 3**. Network Meta-Analysis on Associations of Cardiometabolic Risk Factors and COVID-19 Hospitalization.


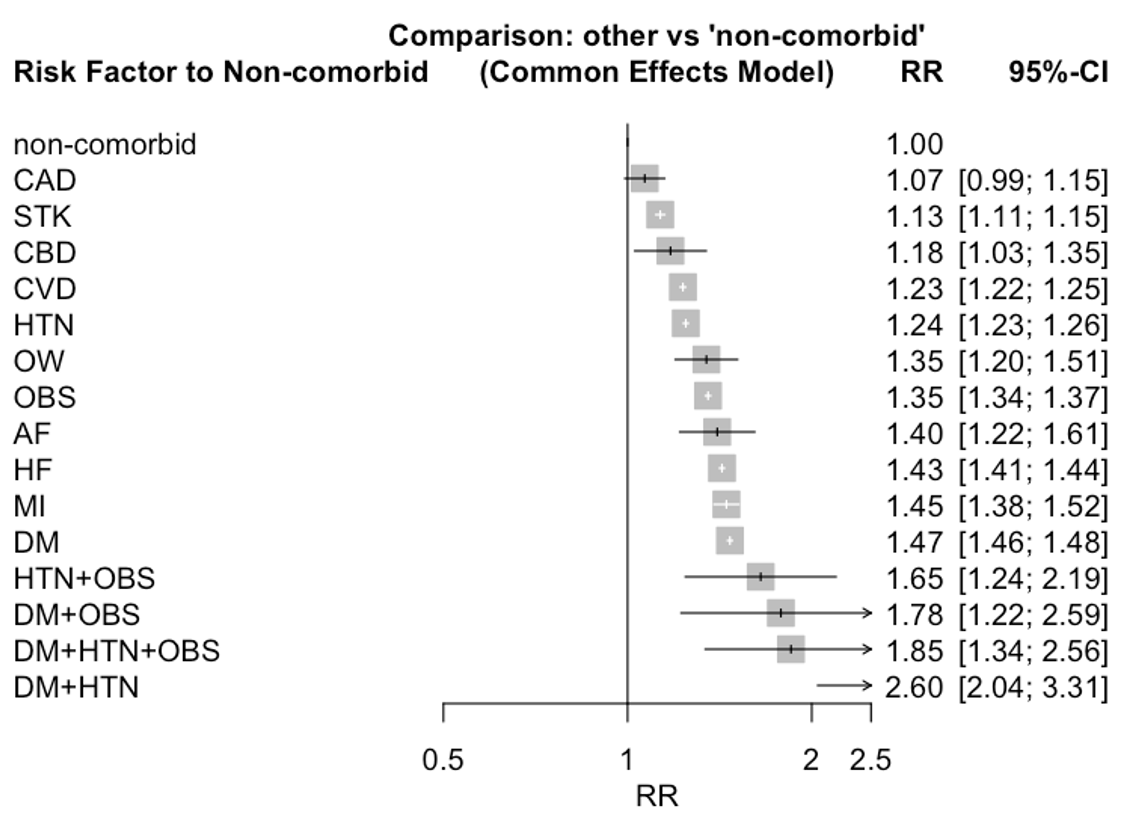


Abbreviations: AF, atrial fibrillation; CAD, coronary artery disease; CBD, cerebrovascular disease; CVD, cardiovascular disease; DM, diabetes mellitus; HF, heart failure; HTN, hypertension; MI, myocardial infarction; non-comorbid, absence of any cardiometabolic comorbidity; OBS, obesity; OW, overweight; STK, stroke.

**Supplementary Figure 4**. Network Meta-Analysis on Associations of Cardiometabolic Risk Factors and COVID-19 Severity.


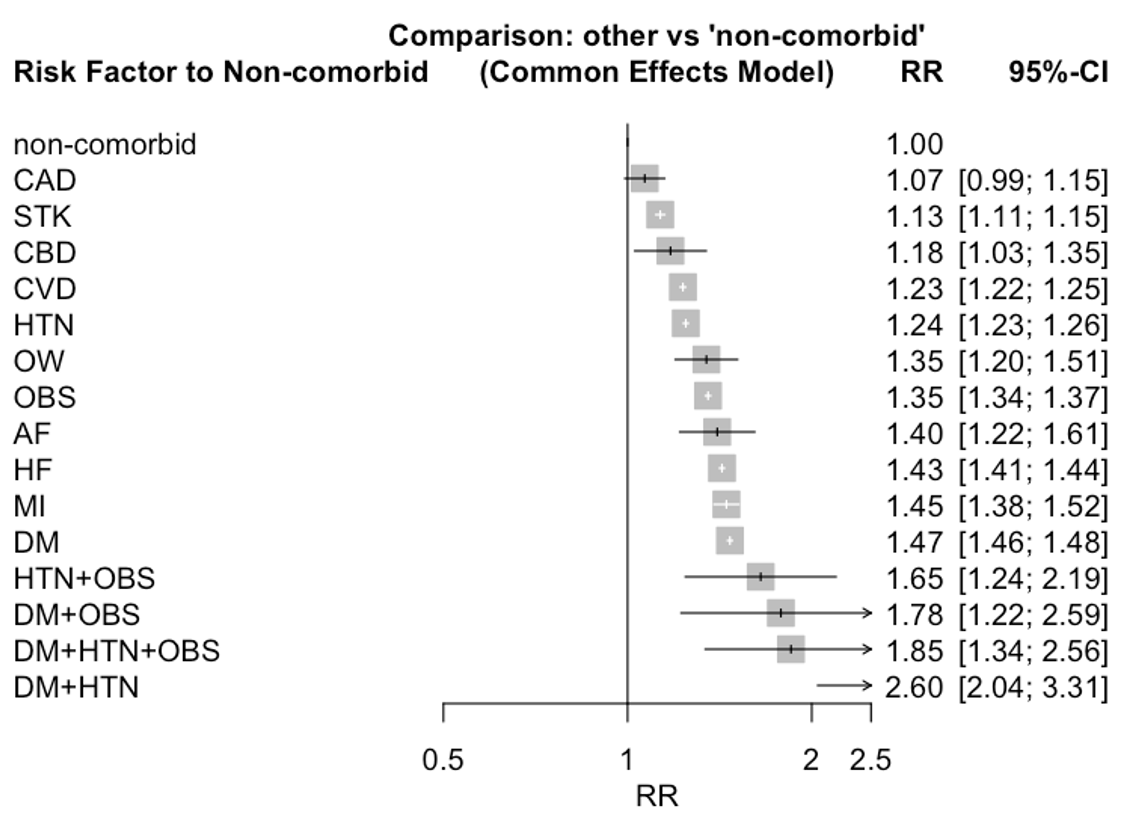


Abbreviations: AF, atrial fibrillation; CAD, coronary artery disease; CBD, cerebrovascular disease; CVD, cardiovascular disease; DM, diabetes mellitus; HF, heart failure; HTN, hypertension; MI, myocardial infarction; non-comorbid, absence of any cardiometabolic comorbidity; OBS, obesity; OW, overweight; STK, stroke.

**Supplementary Figure 5**. Network Meta-Analysis on Associations of Cardiometabolic Risk Factors and COVID-19 Mortality.


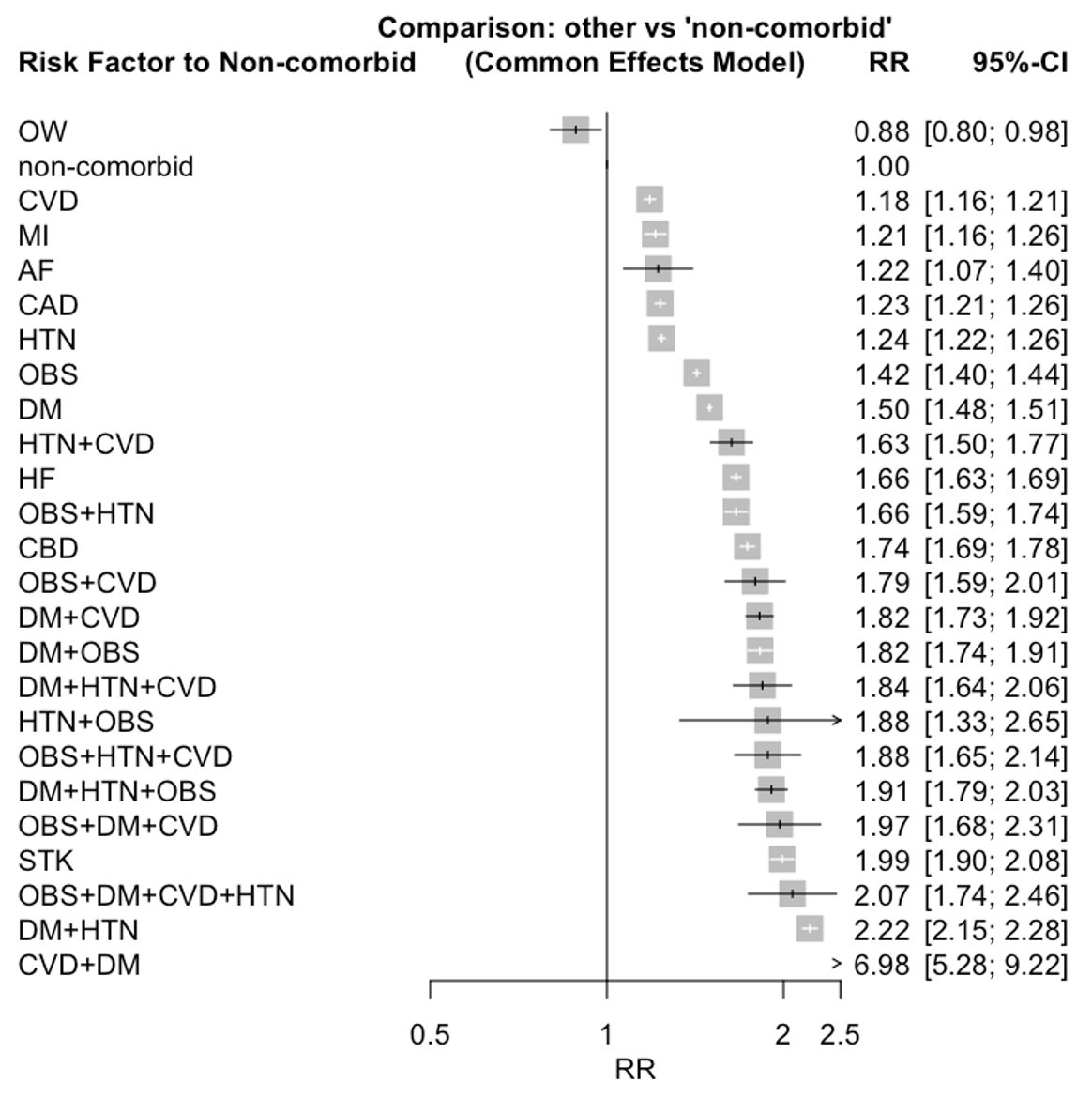


Abbreviations: AF, atrial fibrillation; CAD, coronary artery disease; CBD, cerebrovascular disease; CVD, cardiovascular disease; DM, diabetes mellitus; HF, heart failure; HTN, hypertension; MI, myocardial infarction; non-comorbid, absence of any cardiometabolic comorbidity; OBS, obesity; OW, overweight; STK, stroke.
